# Optical Alignment and Optical Orientation of Excitons in CdSe/CdS Colloidal Nanoplatelets

**DOI:** 10.3390/nano13172402

**Published:** 2023-08-24

**Authors:** Olga O. Smirnova, Ina V. Kalitukha, Anna V. Rodina, Grigorii S. Dimitriev, Victor F. Sapega, Olga S. Ken, Vladimir L. Korenev, Nikolai V. Kozyrev, Sergey V. Nekrasov, Yuri G. Kusrayev, Dmitri R. Yakovlev, Benoit Dubertret, Manfred Bayer

**Affiliations:** 1 Ioffe Institute, Russian Academy of Sciences, 194021 St. Petersburg, Russia; 2 Experimentelle Physik 2, Technische Universität Dortmund, 44221 Dortmund, Germany; 3 Laboratoire de Physique et d’Étude des Matériaux, ESPCI, CNRS, 75231 Paris, France

**Keywords:** excitons, spins, optical orientation, optical alignment, nanoplatelets

## Abstract

Optical alignment and optical orientation of excitons are studied experimentally on an ensemble of core/shell CdSe/CdS colloidal nanoplatelets. Linear and circular polarization of photoluminescence during resonant excitation of excitons is measured at cryogenic temperatures and with magnetic fields applied in the Faraday geometry. The developed theory addresses the optical alignment and optical orientation of excitons in colloidal nanocrystals, taking into account both bright and dark exciton states in the presence of strong electron–hole exchange interaction and the random in-plane orientation of nanoplatelets within the ensemble. Our theoretical analysis of the obtained experimental data allows us to evaluate the exciton fine structure parameters, the *g*-factors, and the spin lifetimes of the bright and dark excitons. The optical alignment effect enables the identification of the exciton and trion contributions to the emission spectrum, even in the absence of their clear separation in the spectra.

## 1. Introduction

Colloidal semiconductor nanocrystals have a long history in fundamental research [1,2,3]. They are attracting a lot of attention at present due to their growing applicability across diverse fields, including chemistry, physics, biology, and medicine [4]. Colloidal nanocrystals can be synthesized from a large variety of semiconductor materials. The synthesis allows nanocrystals with different dimensions and shapes, such as zero-dimensional quantum dots, one-dimensional nanorods, and two-dimensional nanoplatelets (NPLs) [5]. The first synthesized CdSe NPLs demonstrated small inhomogeneous broadening [6], observed later for NPLs of different compositions [7,8,9]. Importantly, the strong spatial and dielectric confinement of carriers in CdSe NPLs results in the large exciton binding energy of about 200–300 meV [10], allowing studies of the physics of two-dimensional excitons even at room temperature.

While many works have been devoted to the optical properties of excitons and exciton complexes in colloidal nanocrystals, their spin physics remains less explored. The experimental techniques developed and widely used in spin physics of epitaxially grown semiconductor nanostructures can be readily used for colloidal nanocrystals. For example, spin-flip Raman scattering has allowed one to observe single and double electron spin-flips in CdSe NPLs [11]. Surface-induced magnetism has recently been detected using polarized photoluminescence (PL) in the external magnetic field in CdSe NPLs [12]. However, the properties of colloidal and epitaxial nanostructures can differ considerably due to much stronger confinement of charge carriers in colloidal nanocrystals. The enhanced electron–hole interaction and, thus, significant exciton fine structure energy splitting, potential for photocharging, and random orientation of the colloidal nanocrystals in an ensemble, make studies of their spin physics a new fundamental problem, which calls for developing new theoretical models [13].

Optical orientation and optical alignment spectroscopies are powerful tools for studying the spin physics in solids. They were successfully used for semiconductors and epitaxial semiconductor heterostructures [14,15,16,17,18]. These methods—backed up by theory—contributed greatly to the characterization of electronic excitations in semiconductors: the times of spin dynamics [19,20,21,22], effective *g*-factors of carriers [23], and the parameters of exciton fine structures [24].

Optical orientation is the excitation of states with a certain projection of angular momentum by the incident circularly polarized light. The effect manifests itself in the circular polarization of the subsequent emission, provided by the recombination of spin-oriented carriers or excitons. It can be observed by exciting unbound electron–hole pairs or excitons, as well as trions.

Optical alignment is provided by the excitation of states with a certain direction of a dipole moment by linearly polarized light [14]. The effect manifests itself in linear polarization of the subsequent emission if the correlation (coherence) between the electron and hole spins is preserved. Such correlation is possible only for the recombination of an electron and a hole generated in the same act of absorption of linearly polarized light or under resonant excitation of excitons [25].

In the case of excitons in GaAs-based nanostructures [16], the optical orientation is given by the difference in populations of the bright exciton states with a projection of the total angular momenta, +1 and −1, on the quantization axis, created via the absorption of circularly polarized light. The optical alignment consists of creating a coherent superposition of a pair of optically active states ±1 by linearly polarized light. Both the difference in populations and the coherence of spin states evolve over time under the influence of various interactions. If the optical orientation or alignment of excitons is preserved during their lifetime, the PL is partially polarized. Its polarization can be characterized by the three Stokes parameters in addition to the intensity. An external magnetic field affects both phenomena and, moreover, can induce the conversion between linear and circular polarization, and vice versa [24].

The study of optical orientation and optical alignment in a magnetic field provides comprehensive information on the fine structures of exciton spin levels. In the zero magnetic field, the exchange interaction between an electron and a hole splits the four-fold degenerate quadruplet ground state of the exciton in two-dimensional NPLs with the zinc blende crystal structure into an optically active doublet (bright excitons) and two energetically close optically forbidden singlets (dark excitons) [26], similar to the situation in quantum wells [16]. The exciton localization in the NPL lowers the symmetry of the system, and the optically active doublet splits into two sublevels [27,28,29], which are linearly polarized along two orthogonal directions defined by the in-plane asymmetry of the localizing potential. Knowledge of the fine structure of the exciton (the directions of the main axes and the values of the energy splittings) allows one to infer the symmetry of the nanostructure and the optical selection rules that are important for understanding the radiation pattern of the NPLs. In experiments, an ensemble of NPLs with various in-plane shapes, sizes, and orientations is commonly studied. In this case, the characteristic splittings of the exciton fine structure are indistinguishable in the PL spectrum due to large inhomogeneous broadening. Nevertheless, the exciton fine structure can become evident in the polarization of the exciton PL, even in the absence of sufficient spectral resolution [16,24,28,30].

Colloidal nanocrystals are often charged with electrons or holes. A significant contribution to the optical properties of such nanocrystals comes from three-particle complexes—trions, consisting of two electrons (or two holes) in the spin singlet state and one hole (or one electron). The negative trions are known to be strong in the emission of CdSe-based NPLs [31,32]. Negative photocharging of CdSe/CdS NPLs was also detected in pump–probe Faraday rotation experiments [33]. Importantly, optical orientation can also be observed for trions. As a result, the overlap of the trion PL and the exciton PL in the spectrum complicates the interpretation of optical orientation experiments [34]. Optical alignment becomes an essential signature in this case. There is no correlation between the electron and hole spins in the trion and, therefore, there is no optical alignment effect. Hence, the observation of exciton optical alignment allows one to distinguish between the trion and exciton contributions to the PL even in the absence of a clear spectral separation between excitons and trions.

Optical orientation, optical alignment, and polarization conversion effects were observed in epitaxial nanostructures [35,36]. In colloidal nanocrystals and NPLs, the emission at low temperatures comes mostly from the weak radiative recombination of the dark exciton. The energy relaxation from the bright exciton is provided by an electron or hole spin-flip so that the spin orientation and/or coherence can be lost. We are not aware of an experimental or theoretical study of optical orientation, optical alignment, or polarization conversion of excitons in II–VI or III–V colloidal nanocrystals. Recently, the optical orientation and optical alignment of excitons were reported for CsPbI${}_{3}$ lead halide perovskite nanocrystals, which have a considerably different electronic band structure [37].

Here, we report on the experimental observation of optical alignment and optical orientation of excitons in core/shell CdSe/CdS NPLs. The PL polarization under continuous-wave (cw) excitation was measured in magnetic fields applied in the Faraday geometry. Surprisingly, even at low temperatures, we observe a strong contribution of the bright exciton to the emission and the optical alignment. Even more surprisingly, for strongly resonant excitation conditions, we observe an additional weak contribution to the optical alignment, which can be attributed to the dark exciton. We develop a theory of optical alignment and optical orientation, taking into account the peculiarities of colloidal NPLs: (i) the large electron–hole exchange interaction, resulting in a large bright–dark exciton splitting; (ii) the presence of dark exciton radiative recombination; and (iii) the random in-plane orientation of the NPLs in the ensemble. The analysis of the experimental data allows us to evaluate the anisotropic energy splittings between the exciton states (in the zero magnetic field), the *g*-factors, and the spin lifetimes of the bright and dark excitons.

## 2. Experiment

**Figure 1:**
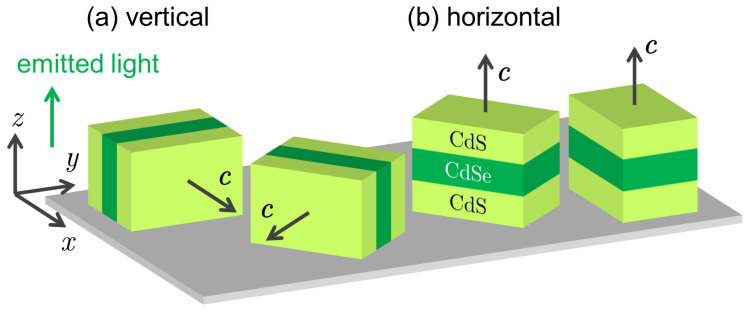
Preferred orientations of the CdSe/CdS NPLs on the substrate. (**a**) Vertically oriented NPLs (standing on their edge on the substrate) with the anisotropic c-axis lying in the substrate plane. (**b**) Horizontally oriented NPLs (lying on the substrate) with the c-axis perpendicular to the substrate plane. Both vertical and horizontal NPLs are randomly oriented in the plane of the sample, which is illustrated by the corresponding pairs of NPLs.

We study NPLs with a 1.2 nm (four monolayers) thick CdSe core and a CdS shell, schematically represented in Figure 1 (for more details, see Section 6). Series of CdSe/CdS NPLs with different shell thicknesses were investigated previously by means of a pump–probe technique, detecting the electron spin coherence at room temperature [33]. In particular, the electron *g*-factor and its dependence on the shell thickness were reported.

In the present paper, we report on low-temperature and temperature-dependent studies of the PL properties and measurements of the optical alignment and optical orientation of excitons. The experimental phenomenology is qualitatively the same for samples with shell thicknesses of 3.1, 5.2, and 9 nm on each side of the CdSe core. We focus on the results for the NPLs with 3.1 nm CdS shell thickness. Selected results for 5.2 and 9 nm CdS shell thicknesses can be found in the Supplementary Materials, Figure S1.

### 2.1. Continuous Wave Polarized Photoluminescence

**Figure 2:**
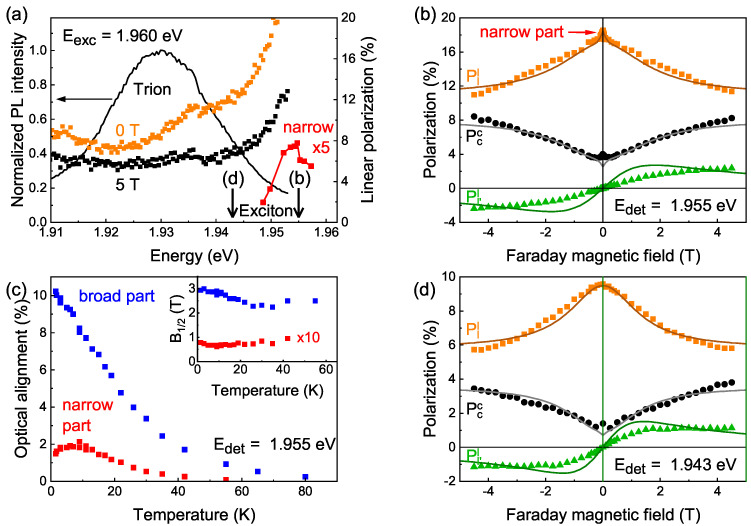
Polarized PL spectroscopy of CdSe/CdS NPLs for resonant excitations with $E_{\mathrm{exc}}=1.960$ eV at T=1.5 K. (**a**) PL spectrum (black line), linear polarization for linearly polarized excitation at zero magnetic field (orange squares) and in B=5 T (black squares), the amplitude of the narrow part of the optical alignment contour (red squares, multiplied by 5). Arrows are pointing at the detection energies for panels (**b**,**d**). (**b**,**d**) Experimental data (symbols) and theoretical calculations (solid lines of corresponding colors) of the linear polarization $P_{l}^{l}$ (orange squares), the rotation of the linear polarization plane $P_{l^{\prime }}^{l}$ (green triangles) and the optical orientation $P_{c}^{c}$ (black circles), measured in the Faraday geometry at (**b**) $E_{\det}=1.955$ eV and (**d**) $E_{\det}=1.943$ eV. (**c**) Temperature dependence of the amplitude of the broad (blue squares) and narrow (red squares) parts of the optical alignment contour. Inset: temperature dependence of their HWHM (denoted as $B_{1/2}$). $B_{1/2}$ of the narrow part is multiplied by 10.

The PL spectrum of the 1.2 nm CdSe core/3.1 nm CdS shell NPLs measured at the temperature of T=1.5 K under laser excitation at $E_{\mathrm{exc}}=1.960$ eV photon energy (Figure 2a) consists of one broad band centered at 1.930 eV. The shape of the PL spectrum remains the same for higher excitation energy, under pulsed instead of cw excitation, and at temperatures increased up to 40 K (see details in Supplementary Materials, Figure S1a). Such a spectrum is typical for CdSe/CdS core/shell NPLs [12,31,38], in contrast to bare core CdSe NPLs, where the PL spectrum has separated exciton and trion lines [32]. In addition to the radiative exciton and trion recombination, the low-energy part of the PL spectrum in CdSe/CdS NPLs can also be affected by so-called shake-up processes. The shake-up process consists of the recombination of the trion or the exciton interacting with resident electrons, leaving one of these electrons in the excited state [39]. The presence of the CdS shell opens up additional traps for the resident electrons so that the fluctuations of the localization energies lead to the broadening of the spectra.

In order to study the effects of (1) optical alignment, (2) rotation of the linear polarization plane, and (3) optical orientation, we measure the three corresponding polarization parameters: (1) the degree of the PL linear polarization along the main axes (*x* and *y* corresponding to horizontal and vertical linear polarization) for linearly polarized laser excitation $P_{l}^{l}$, (2) the degree of the PL linear polarization with axes rotated to the diagonal between the main axes for linearly polarized excitation $P_{l^{\prime }}^{l}$, and (3) the degree of the PL circular polarization for circularly polarized excitation $P_{c}^{c}$. The three parameters are defined as follows:

(1) $$P_{l}^{l}=\frac{I_{x}^{x}-I_{y}^{x}}{I_{x}^{x}+I_{y}^{x}}\;,\;P_{l^{\prime }}^{l}=\frac{I_{x^{\prime }}^{x}-I_{y^{\prime }}^{x}}{I_{x^{\prime }}^{x}+I_{y^{\prime }}^{x}}\;,\;P_{c}^{c}=\frac{I_{+}^{+}-I_{-}^{+}}{I_{+}^{+}+I_{-}^{+}}\;.$$

Here, $I_{x(y)}^{x}$ is the intensity of the horizontally (vertically) polarized component of the PL for horizontally polarized excitation, $I_{x^{\prime }\left(y^{\prime }\right)}^{x}$ is the intensity of the 45${}^{\circ }$ ($-45^{\circ }$) polarized component of the PL for the horizontally polarized excitation, and $I_{+(-)}^{+}$ is the intensity of the $\sigma^{+}$ ($\sigma^{-}$)-polarized component of the PL for the $\sigma^{+}$-polarized excitation.

These three parameters are studied in magnetic fields of up to 5 T applied in the Faraday geometry with B‖k, where k is the wave vector of the emitted light (marked by the green arrow in Figure 1). For more details on these polarization measurements, see Section 6.

In our study, we find all three effects—optical alignment, rotation of the linear polarization plane, and optical orientation—in the studied NPLs for resonant excitation of the exciton at $E_{\mathrm{exc}}=1.960$ eV. While the $P_{c}^{c}$ changes weakly across the PL spectrum (see Supplementary Materials, Section S1), the $P_{l}^{l}$ depends significantly on the detection energy. Figure 2a shows the spectrum of the linear polarization of the PL in zero magnetic field (orange squares) and in the Faraday magnetic field B=5 T (black squares) for the linearly polarized cw excitation in comparison to the PL spectrum (black). The optical alignment effect is even, with respect to the magnetic field. Therefore, it can be quantified as the difference between the two linear polarization spectra: the optical alignment is minimal around the detection energy $E_{\det}=1.920$ eV and increases toward the high-energy part of the spectrum up to 10%. This spectral dependence suggests that the PL at the high-energy part of the spectrum is contributed to by the exciton recombination, which is verified by time-resolved PL measurements (Section 2.3). The PL around its maximum is mostly contributed to by negatively charged trions, composed of two electrons and one hole, because optical alignment is not expected for singlet trions or any fermion quasi-particles.

The linear polarization $P_{l}^{l}$ contains an additional contribution besides the optical alignment. The spectrum in B=5 T roughly represents this constant (with respect to the magnetic field) contribution to the $P_{l}^{l}$, originating from a structural anisotropy of the NPL ensemble, namely the linear polarization of the exciton and trion emissions of the vertical NPLs. The vertical NPLs emit linearly polarized light with k‖z⊥c for both linearly and circularly polarized excitations because the recombination involving the heavy holes is always polarized perpendicular to the c-axis. Linearly polarized light excites vertical NPL selectively, depending on their orientation; therefore, the linear polarization of the PL is preserved in the ensemble measurements (linear polarization memory effect). However, in the case of circularly polarized excitation, the linear polarization of the PL vanishes due to the averaging over the randomly oriented vertical NPLs shown in Figure 1a.

Figure 2b shows the dependence of all three polarization parameters defined in Equation (1) on the magnetic field applied in the Faraday geometry. The measurements were performed at the detection energy $E_{\det}=1.955$ eV. In this section, we use simple fit functions (Lorentzian or bi-Lorentzian curves plus a constant) to estimate the characteristic parameters of the $P_{l}^{l}$ and $P_{c}^{c}$ curves, the amplitudes and half widths at half maxima (HWHMs, denoted as $B_{1/2}$), although they do not have a straightforward physical meaning in the case of randomly in-plane-oriented NPLs. In the theory section, we present a model that allows us to estimate the physical parameters of the excitons in NPLs.

The linear polarization $P_{l}^{l}$ (orange squares) consists of a constant contribution of the order of 10% (due to vertical NPLs) and the optical alignment, depending on the magnetic field: the broad part of the contour that has an HWHM of about 3 T and the narrow one has a HWHM of less than 0.1 T. The spectral dependence of the amplitude of the narrow part is shown in Figure 2a with red symbols. The narrow part of the optical alignment contour is resonant with respect to the laser excitation energy. Its amplitude reaches a maximum when the PL is detected at about 5 meV below the laser energy, regardless of the particular laser energy (within the resonant exciton excitation), and rapidly vanishes with a variation in the detection energy.

Figure 2c shows the temperature dependence of the broad and the narrow parts of the optical alignment. The broad part amplitude is 10% at T=1.5 K; it decreases with growing temperature and disappears above 80 K. The narrow part of the optical alignment contour has an amplitude of 1.5% at 2 K. It rises to 2% around T=5 K, and then decreases (similar to the broad part) and disappears above 60 K. Dependences of the HWHMs are shown in the inset of Figure 2c. For both sections, we note a slight temperature dependence.

The rotation of the linear polarization plane $P_{l^{\prime }}^{l}\left(B\right)$ (green triangles in Figure 2b), has a nonlinear antisymmetric dependence on the magnetic field applied in the Faraday geometry. The value of the linear polarization degree in the magnetic field of 4 T is 3%. The optical orientation $P_{c}^{c}\left(B\right)$ (black circles in Figure 2b) increases in amplitude up to 6% in the magnetic field. The HWHM of the optical orientation is 3 T, which corresponds to the broad part of the optical alignment contour and to the magnetic field at which the rotation of the linear polarization plane reaches saturation.

The magnetic field dependences of all three polarization parameters ($P_{l}^{l}$, $P_{l^{\prime }}^{l}$, and $P_{c}^{c}$) measured at the detection energy $E_{\det}=1.943$ eV are presented in Figure 2d. This energy is 17 meV lower than the excitation energy, thus the narrow part of the optical alignment contour is absent here. The amplitudes of the optical alignment and optical orientation curves decrease with respect to the more resonant detection energy $E_{\det}=1.955$ eV to 4.5% and 3%, respectively. The value of the linear polarization degree in the magnetic field decreases to 1.3% in 4 T. The HWHMs of the optical alignment and optical orientation correspond to the saturation field of the rotation of the linear polarization plane and are equal to 2 T, which is smaller compared to the 3 T at $E_{\det}=1.955$ eV.

### 2.2. Raman Scattering

**Figure 3:**
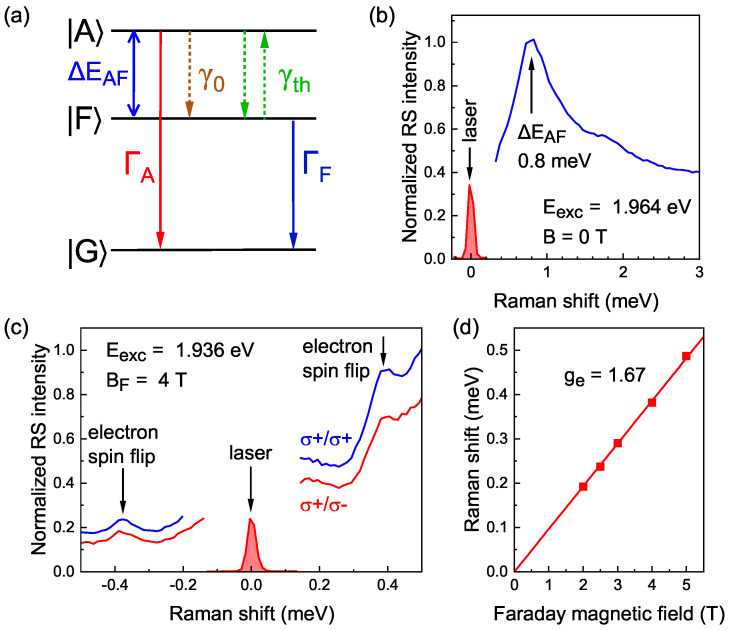
(**a**) Scheme of the low energy levels of the bright |A〉(±1) and the dark |F〉(±2) excitons split by $\Delta E_{AF}$ and the ground state |G〉. $\Gamma_{A},\Gamma_{F}$ are the radiative recombination rates, $\gamma_{0}$ is the relaxation rate at zero temperature, $\gamma_{\mathrm{th}}$ is the temperature-induced relaxation. (**b**) Raman scattering (RS) spectrum for excitation energy $E_{\mathrm{exc}}=1.964$ eV at zero magnetic field at T=1.5 K. Laser spectrum is shown by the red filled curve. (**c**) SFRS spectra measured for $E_{\mathrm{exc}}=1.936$ eV excitation energy in the Faraday magnetic field $B_{F}=4$ T at T=1.5 K. Both spectra are measured using $\sigma^{+}$ polarized excitation. Blue (red) spectrum gives the $\sigma^{+}$($\sigma^{-}$) polarized component of the measured signal, correspondingly. (**d**) Experimental dependence of the Raman shift of the electron spin-flip line on the magnetic field applied in the Faraday geometry (symbols) and the linear fit to it (line).

Raman scattering spectroscopy in the zero magnetic field allows one to measure the energy splitting between the bright (optically allowed, A) and the dark (optically forbidden, F) exciton states $\Delta E_{AF}$ (Figure 3a) caused by the electron–hole exchange interaction. Figure 3b shows a Raman spectrum at $E_{\mathrm{exc}}=1.964$ eV laser excitation energy, measured at T=1.5 K. The spectrum demonstrates a line at the energy of 0.8 meV. This line corresponds to the emission of the dark exciton after energy relaxation from the initially excited bright state. Thus, the energy splitting between the bright and dark exciton states is $\Delta E_{AF}=0.8$ meV.

The determination of the electron *g*-factor ($g_{e}$) is conducted by means of spin-flip Raman scattering (SFRS) spectroscopy in the magnetic field. SFRS spectra in co- and cross-circular polarizations measured for resonant excitation at $E_{\mathrm{exc}}=1.936$ eV in the Faraday magnetic field $B_{F}=4$ T are shown in Figure 3c. Both the Stokes (with positive Raman shifts) and anti-Stokes (with negative Raman shifts) ranges of the spectra show a line in the energy shift of 0.38 meV. This line is attributed to the spin-flip of the electron. Figure 3d shows the magnetic field dependence of the Raman shift of this electron spin-flip line. Its approximation with linear Zeeman energy scaling, $\mu_{B}g_{e}B_{F}$, where $\mu_{B}$ is the Bohr magneton, gives the *g*-factor of electron $g_{e}=1.67$. This value is close to the 1.59 value determined for this sample at room temperature in Ref. [33].

### 2.3. Time-Resolved Photoluminescence

The analysis of the temperature-dependent and magnetic-field-dependent PL dynamics allows us to prove that the high energy part of the PL spectrum (Figure 2a) is contributed mostly by exciton recombination. At the same time, trion recombination determines the dynamics at the PL maximum.

The PL spectra for pulsed excitation (both resonant and nonresonant) at T=1.5 K consist of a broad line with a maximum of 1.930 eV, similar to the cw PL spectrum, shown in the Supplementary Materials, Figure S1a.

**Figure 4:**
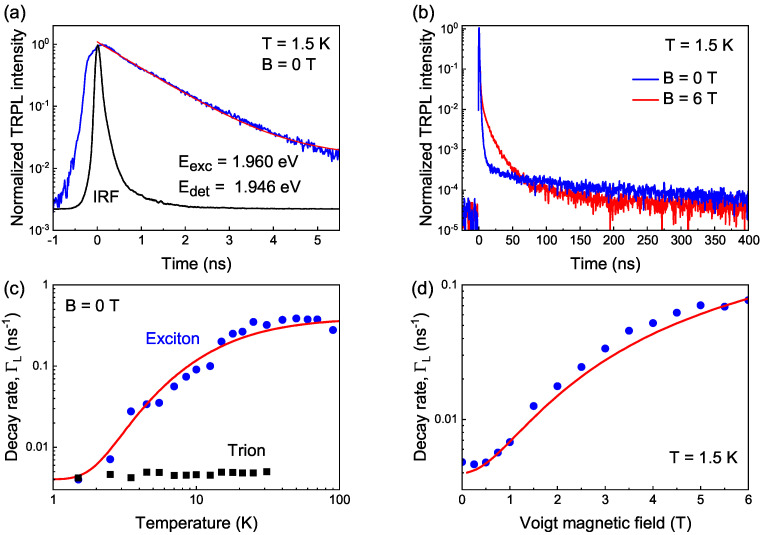
Time-resolved PL of CdSe/CdS NPLs for resonant excitation with $E_{\mathrm{exc}}=1.960$ eV photon energy. (**a**) PL dynamics at short times (blue) with a monoexponential fit (red). Black curve represents the instrumental response function (IRF). (**b**) PL dynamics in different magnetic fields applied in the Voigt geometry. (**c**) Experimental temperature dependence of the trion decay rate (black squares) and the exciton decay rate $\Gamma_{L}$ (blue circles) compared with the one calculated by Equation (S6) (red line). (**d**) Experimental dependence of $\Gamma_{L}$ on the magnetic field (blue squares) applied in the Voigt geometry and the theoretical curve (red line, Equation (S7)). The fit parameters for the theoretical curves are presented in the text.

The dynamics of the PL obtained for resonant pulsed excitation with $E_{\mathrm{exc}}=1.960$ eV, detected at the high-energy part of the PL spectrum ($E_{\det}=1.946$ eV), are shown in Figure 4a,b. These excitation–detection conditions correspond to the exciton PL. The exciton PL decay has a short- and a long-lived component with the rates $\Gamma_{S}$ and $\Gamma_{L}$, respectively. The fast decay (within nanoseconds) at T=1.5 K is shown in Figure 4a. It corresponds to the bright exciton dynamics [26] with the lifetime $\tau_{A}=0.9$ ns. This lifetime is determined by the radiative recombination of the bright exciton with the $\Gamma_{A}$ rate and the relaxation from the bright to the dark exciton state with the $\gamma_{0}$ rate so that $\tau_{A}=\Gamma_{S}^{-1}={\left(\Gamma_{A}+\gamma_{0}\right)}^{-1}$. The slow decay in the zero magnetic field is shown in Figure 4b (blue curve). This long component is observed due to the significant population of the dark exciton state and its nonzero oscillator strength, caused by a weak admixture of the bright exciton state. At T=1.5 K, the slow component is determined solely by the dark exciton recombination rate $\Gamma_{L}=\Gamma_{F}$, so that the dark exciton lifetime is given by $\tau_{F}=\Gamma_{L}^{-1}=250$ ns.

The PL dynamics were measured in the temperature range of up to 90 K (typical data are shown in Figure S2a). The excitation–detection conditions corresponding to the resonant excitation of the exciton PL are the same for all temperatures since the PL spectrum practically does not shift or change shape with temperature (Figure S1a). Figure 4c shows the temperature dependence of the decay rate, $\Gamma_{L}$. Such behavior is typical for exciton recombination [26], and is caused by the temperature-induced redistribution of the bright and dark exciton state populations (see Supplementary Materials Section S2), in combination with the phonon-assisted relaxation rate $\gamma_{\mathrm{th}}=\gamma_{0}N_{B}$ (Figure 3a), where $N_{B}\left(E_{\mathrm{ph}}\right)=1/\left(\exp\left(E_{\mathrm{ph}}/k_{B}T\right)-1\right)$ is the phonon occupation, $E_{\mathrm{ph}}$ is the phonon energy, and $k_{B}$ is the Boltzmann constant. The analysis of $\Gamma_{L}\left(T\right)$ allows us to determine the exciton parameters [26], including the bright and dark exciton recombination rates, as well as the relaxation rates and the energy splitting $\Delta E_{AF}$ (see Supplementary Materials, Section S2). From the fit to the $\Gamma_{L}\left(T\right)$ dependence with Equation (S6), we obtain the exciton rates $\gamma_{0}=0.31$ ns${}^{-1}$, $\Gamma_{A}=0.8$ ns${}^{-1}$, and $\Gamma_{F}=0.004$ ns${}^{-1}$, using the value $\Delta E_{AF}=0.8$ meV determined from the Raman scattering measurements.

The PL decay depends also on the magnetic field applied to the Voigt geometry, when B⊥k (Figure 4d). The dependence $\Gamma_{L}\left(B\right)$ is caused by the field-induced mixing of the bright and dark exciton states. It is well described by Equation (S7) with the same exciton rates, exciton splitting, and the electron *g*-factor $g_{e}=1.67$ obtained from the spin-flip Raman measurements.

The temperature series of the PL decay are similarly recorded for the nonresonant excitation energy $E_{\mathrm{exc}}=2.380$ eV (Figure S2c) with $E_{\det}=1.952$ eV. The $\Gamma_{L}\left(T\right)$ dependence coincides with the case of resonant excitation (Figure S2d). Thus, independent of the excitation energy, and in the range of detection energies, the PL around 1.946 eV (where the effect of optical alignment is observed, see Figure 2a), is contributed to mostly by exciton recombination. In contrast, the trion PL dynamics at 1.930 eV (Figure S2b) do not depend on the temperature (Figure 4c). This is typical for trions that do not have a dark state [31,32].

The electron–hole exchange interaction, as well as the direct Coulomb interaction in NPLs, are enhanced by the strong spatial confinement along the c-axis and by the dielectric contrast between the NPL and the surrounding ligands [10,26]. As a result, in 4-monolayer bare core CdSe NPLs, $\Delta E_{AF}$ and the exciton binding energy are as large as 4.5 meV and 270 meV, respectively [10]. The presence of the CdS shell decreases the electron–hole overlap because of the leakage of a significant part of the electron wave function into the shell. It also decreases the dielectric contrast. This results in a reduction of $\Delta E_{AF}$ to 0.8 meV in the studied CdSe/CdS NPLs and the respective decrease in the exciton binding energy. However, both remain large enough to exclude (i) the excitation of a coherent superposition of the bright and dark exciton states in an NPL and (ii) the excitation of unbound electron–hole pairs at the resonant excitation energy, where the effects of optical alignment and optical orientation are observed.

Thus, we observe both the effects of optical alignment and optical orientation at the high-energy edge of the PL spectrum of an ensemble of core/shell CdSe/CdS NPLs. Our detailed studies of these effects and of the time-resolved PL decay allow us to confirm that at the high-energy edge of the spectrum, the PL mostly comes from exciton recombination. Further, we will develop a theoretical analysis for the optical alignment and the optical orientation of excitons in NPLs with large bright–dark splitting.

## 3. Theory

**Figure 5:**
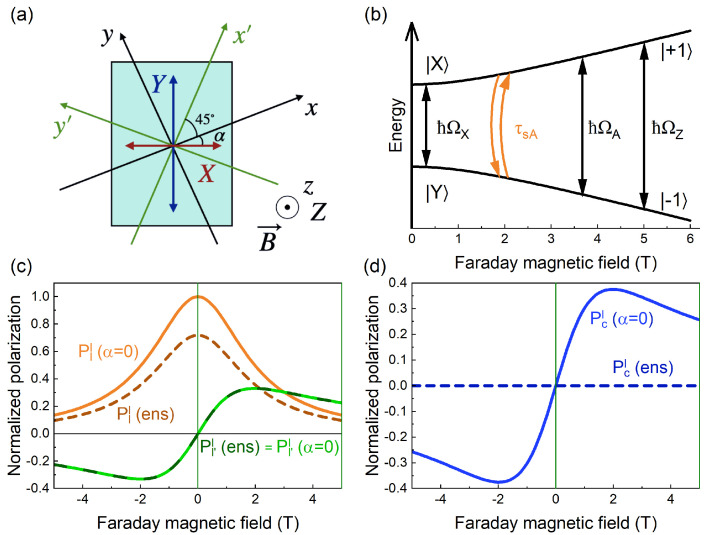
(**a**) Scheme of a single NPL with its own axes X,Y and the laboratory frames: main axes x,y,z and the axes $x^{\prime },y^{\prime }$ rotated by $45^{\circ }$ around the z-axis, the magnetic field is applied in the Faraday geometry. (**b**) Representation of the conversion of the linearly polarized states |X〉,|Y〉 of the bright exciton, split by the energy $\hbar \Omega_{X}$, into the circularly polarized components |+1〉,|−1〉 in the case of $g_{A}>0$. A similar transformation takes place for the dark exciton states |FX〉,|FY〉,|+2〉,|−2〉, and is shown in Figure S3c. Calculated (**c**) optical alignment $P_{l}^{l}$ (orange) and rotation of the linear polarization $P_{l^{\prime }}^{l}$ (green), (**d**) linear polarization conversion into circular $P_{c}^{l}$ (blue), normalized to the amplitude of optical alignment $P_{l}^{l}\left(\alpha =0\right)$. Calculations are shown for the case of α=0 individual and in-plane-oriented NPLs (solid lines) and for a randomly oriented ensemble of NPLs (designated as ens, dashed lines).

Here, we develop a theory of optical alignment and optical orientation of excitons, taking into account the peculiarities of the colloidal NPLs: the large bright–dark exciton splitting with activated radiative emissions from the dark exciton and the presence of the differently oriented NPLs on the substrate. First, we consider the NPLs, oriented horizontally on the substrate as schematically shown in Figure 1b, taking into account their in-plane random orientation. The c-axis of all NPLs under consideration is, thus, directed perpendicular to the substrate and along the [001] crystallographic direction. Generally, the NPL edges can be directed either along the [100] and [010] or along [110] and $\left[1\bar{1}0\right]$ directions [40], and be of different lengths. The in-plane anisotropy of the studied NPLs is not large [33], however, it is present. Therefore, in addition to what is already shown in Figure 1, the laboratory coordinate system with axes x,y,z, we introduce a second coordinate frame related to the NPL with axes X,Y along the NPL edges and axis *Z* directed along c, as shown in Figure 5a. For the normal direction of light propagation k‖z‖Z, the vector of the light polarization $e=(e_{x},e_{y},0)$ is always in the NPL plane. However, the X,Y axes of the NPL frame might be rotated by the angle α with respect to the x,y axes of the laboratory system.

### 3.1. Bright and Dark Exciton Contributions to the PL Polarization

The band-edge exciton states in CdSe-based NPLs comprise bright and dark excitons (for more details see Supplementary Materials, Section S2). These excitons are formed from electrons with spin projection $s_{Z}=\pm 1/2$ on the NPL c-axis and heavy holes with spin projection $j_{Z}=\pm 3/2$. In the absence of an external magnetic field and any anisotropy-related splittings, the bright (A) and dark (F) excitons are given by the two-fold degenerate |±1〉 states described by the wave function $\Psi_{m_{A}}$ with spin projections $m_{A}=s_{Z}+j_{Z}=\pm 1$ and the two-fold degenerate |±2〉 states described by the wave function $\Psi_{m_{F}}$ with spin projections $m_{F}=s_{Z}+j_{Z}=\pm 2$. The bright exciton states with $m_{A}=\pm 1$ can absorb and emit circularly polarized light. The dark exciton states $m_{F}=\pm 2$ interact with light due to the admixture of the bright $m_{A}=\pm 1$ states, caused by small perturbations (interactions with phonons or internal exchange fields) [41].

In the absence of an external magnetic field, the bright (dark) exciton states are split by $\hbar \Omega_{X}$ ($\hbar \Omega_{FX}$) into linearly polarized dipoles |X〉,|Y〉 (and the analogously composed states |FX〉,|FY〉) and described by the wave functions

(2) $$\begin{array}{c} \Psi_{X}=\frac{\Psi_{+1}+\Psi_{-1}}{\sqrt{2}}\;,\;\Psi_{Y}=-i\frac{\Psi_{+1}-\Psi_{-1}}{\sqrt{2}}\;, \end{array}$$

(3) $$\begin{array}{c} \Psi_{FX}=\frac{\Psi_{+2}+\Psi_{-2}}{\sqrt{2}}\;,\;\Psi_{FY}=-i\frac{\Psi_{+2}-\Psi_{-2}}{\sqrt{2}}\;. \end{array}$$

The anisotropic splitting $\hbar \Omega_{X}$ is associated with the long-range electron–hole exchange interaction in the presence of anisotropy of the NPL shape in the plane [29,42]. The splitting between the dark exciton states $\hbar \Omega_{FX}$ has a different nature: even without an in-plane anisotropy, it can originate from the cubic anisotropy contribution to the short-range electron–hole exchange interaction ∼$\sum_{\alpha =X,Y,Z}\sigma_{\alpha }J_{\alpha }^{3}$, where σ is a pseudovector composed of the Pauli matrices and J is the pseudovector of matrices for the angular momentum 3/2 [16]. The fine structure of the band-edge exciton in the presence of anisotropic splitting without an external magnetic field is shown schematically in Figure S3b.

The Hamiltonian, taking into account the electron–hole exchange terms and the Zeeman field-induced term for B‖z for the exciton wave function in the $\left\{\Psi_{+1},\Psi_{-1},\Psi_{+2},\Psi_{-2}\right\}$ basis, has the following matrix form:

(4) $$H_{AF}=\left(\begin{array}{cc} H_{A} & 0\\ 0 & H_{F} \end{array}\right)=\frac{\hbar }{2}\left(\begin{array}{cccc} \Omega_{Z} & \Omega_{X} & 0 & 0\\ \Omega_{X} & -\Omega_{Z} & 0 & 0\\ 0 & 0 & -2\Delta E_{AF}/\hbar +\Omega_{FZ} & \Omega_{FX}\\ 0 & 0 & \Omega_{FX} & -2\Delta E_{AF}/\hbar -\Omega_{FZ} \end{array}\right),$$

where $\hbar \Omega_{Z}=g_{A}\mu_{B}B$, $\hbar \Omega_{FZ}=g_{F}\mu_{B}B$, $g_{A(F)}$—the bright (dark) exciton *g*-factors. The Hamiltonian in Equation (4) does not include any perturbations that can directly mix the bright and dark exciton states. Therefore, the eigenstates of this Hamiltonian, $\Psi_{A}^{\pm }$ and $\Psi_{F}^{\pm }$, comprise only linear combinations of the functions $\Psi_{\pm 1}$ and $\Psi_{\pm 2}$, respectively, with the energy eigenvalues $E_{A}^{\pm }\left(B\right)$ and $E_{F}^{\pm }\left(B\right)$:

(5) $$\begin{array}{ccc} E_{A}^{\pm } & = & \pm \frac{1}{2}\hbar \Omega_{A}=\pm \frac{1}{2}\hbar \sqrt{\Omega_{X}^{2}+\Omega_{Z}^{2}}\;, \end{array}$$

(6) $$\begin{array}{ccc} E_{F}^{\pm } & = & -\Delta E_{AF}\pm \frac{1}{2}\hbar \Omega_{F}\\ & = & -\Delta E_{AF}\pm \frac{1}{2}\hbar \sqrt{\Omega_{FX}^{2}+\Omega_{FZ}^{2}}\;. \end{array}$$

The exciton energy level structure and its evolution in the magnetic field are shown in Supplementary Materials (Section S2, Figure S3). The modification of the bright exciton states in an external magnetic field is shown on a larger scale in Figure 5b.

The polarization of light emitted by excitons can be described using the spin density matrix formalism [24,43]. The splitting between the bright and dark exciton $\Delta E_{AF}$∼0.8 meV is much larger than $\hbar /\tau_{A}$. This allows us to neglect the non-diagonal block terms of the density matrix $\rho_{AF,m_{A}m_{F}},\rho_{AF,m_{F}m_{A}}$, and to consider the block-diagonal density matrix for the four exciton states

(7) $$\rho_{AF}=\left(\begin{array}{cc} \rho_{A} & 0\\ 0 & \rho_{F} \end{array}\right)\;,$$

where the 2×2 density matrices $\rho_{A}$ and $\rho_{F}$ characterize the isolated two-level systems of the bight and dark exciton states $\left\{\Psi_{+1},\Psi_{-1}\right\}$ and $\left\{\Psi_{+2},\Psi_{-2}\right\}$, respectively. They can be expressed with the help of the bright and dark exciton-averaged three-dimensional pseudospins $S_{A}$ and $S_{F}$, as

(8) $$\rho_{A(F)}=N_{A(F)}\left(\frac{1}{2}+\sigma \cdot S_{A(F)}\right).$$

Here, $N_{A}=\rho_{+1,+1}+\rho_{-1,-1}=|\Psi_{+1}{|}^{2}+{\left|\Psi_{-1}\right|}^{2}$ and $N_{F}=\rho_{+2,+2}+\rho_{-2,-2}={\left|\Psi_{+2}\right|}^{2}$ + $|\Psi_{-2}{|}^{2}$ are the bright and dark exciton populations. To simplify the following consideration, hereafter, we consider only the case of cw excitation, in which $N_{A}$ and $N_{F}$ can be found as the steady-state solutions of the corresponding kinetic equations, as described in Supplementary Materials (see Section S2 and Equation (S3)).

The direct absorption of light by the dark exciton states can be safely neglected so that the exciting light contributes only to the bright exciton pseudospin $S_{A}^{0}$. For an NPL with axes X,Y, rotated around the laboratory axis *z* by the angle α, the laser-induced $S_{A}^{0}$ is related to the exciting light polarization as follows:

(9) $$\begin{array}{c} S_{AX}^{0}=\frac{\gamma_{l}}{2}P_{0}^{l}\cos\left(2\alpha \right),\\ S_{AY}^{0}=\frac{\gamma_{l}}{2}P_{0}^{l}\sin\left(2\alpha \right),\\ S_{AZ}^{0}=\gamma_{c}P_{0}^{c}\;. \end{array}$$

Here, $P_{0}^{l(c)}$ is the polarization degree of the linearly (circularly) polarized exciting light and the parameters $\gamma_{l}\leq 1$ and $\gamma_{c}\leq 1$ account for the possible loss of polarization during relaxation in the case when the excitation is not exactly resonant. In the latter case, a dark exciton state population $N_{F}$ can also be created, however, its polarization will be completely lost during the relaxation processes ($\gamma_{l}=0$ and $\gamma_{c}=0$ for the dark exciton).

Even without the direct light absorption by the dark exciton states, their contribution to the PL is important at low temperatures due to their significant population. Accordingly, the total emission intensity from the NPLs $I=I_{A}+I_{F}$ comprises the intensities of the bright exciton $I_{A}=\Gamma_{A}^{r}N_{A}$ and the dark exciton $I_{F}=\Gamma_{F}^{r}N_{F}$, where $\Gamma_{A(F)}^{r}=\eta_{A(F)}\Gamma_{A(F)}$ is the radiative recombination rate, and $\eta_{A(F)}$ is the bright (dark) exciton radiative recombination efficiency.

Then, the polarization of light emitted by the excitons from a single horizontally oriented NPL with axes X,Y can be represented as follows:

(10) $$\begin{array}{c} P_{C}=i\left(e_{X}e_{Y}^{*}-e_{X}^{*}e_{Y}\right)=AP_{CA}+FP_{CF}\;,\\ P_{L}=|e_{X}{|}^{2}-{\left|e_{Y}\right|}^{2}=AP_{LA}+FP_{LF},\\ P_{L^{\prime }}=e_{X}e_{Y}^{*}+e_{X}^{*}e_{Y}=AP_{L^{\prime }A}+FP_{L^{\prime }F}. \end{array}$$

Here, $e_{X}$ and $e_{Y}$ are the projections of the emitted light polarization vector e on the X,Y axes, $A=I_{A}/\left(I_{A}+I_{F}\right)$ and $F=I_{F}/\left(I_{A}+I_{F}\right)$ characterize the contributions from the bright and dark exciton, correspondingly. For resonant excitation of the bright exciton, $A/F=\eta_{A}\Gamma_{A}/\eta_{F}\gamma_{0}$ at a low temperature.

The partial polarizations of the bright exciton emission are related to its averaged pseudospin components, as $P_{CA}=2S_{AZ}$, $P_{LA}=2S_{AX}$, and $P_{L^{\prime }A}=2S_{AY}$. For the dark exciton, the polarization of its emission depends on the particular perturbation resulting in the admixture of the bright exciton states. Such a perturbation, allowing the mixture of the electron spin-up and spin-down states can be caused by the exchange interaction with some paramagnetic centers, for example, dangling bond spins at the NPL surface [12,41]. In this case, the m=±2 states inherit the circular polarization selection rules from the m=±1 states and $P_{CF}=2S_{FZ}$. However, if the in-plane internal exchange field is randomly oriented in each NPL, the emission from the |FX〉 and |FY〉 states is not linearly polarized. Contrarily, if the internal exchange field is predominantly oriented along the *X* or *Y* direction due to the NPL shape anisotropy, the emissions from the |FX〉 and |FY〉 states are also linearly polarized so that $P_{LF}=\pm 2S_{FX}$ [44].

In the laboratory frame, the measured circular polarization, $P_{c}=P_{C}$, as well as the total intensity, *I*, do not depend on the NPL orientation. In contrast, the linear polarization depends on the orientation of the NPL rotated around the laboratory axis *z* by the angle α (Figure 5a), and can be found as follows

(11) $$\begin{array}{c} P_{l}\left(\alpha \right)=P_{L}\cos\left(2\alpha \right)+P_{L^{\prime }}\sin\left(2\alpha \right),\\ P_{l^{\prime }}\left(\alpha \right)=-P_{L}\sin\left(2\alpha \right)+P_{L^{\prime }}\cos\left(2\alpha \right). \end{array}$$

Thus, to describe the effects of optical orientation and optical alignment of excitons, we must first find the components of the bright and the dark exciton pseudospins in an external magnetic field.

### 3.2. Pseudospin Components in the Magnetic Field in the Faraday Geometry

The dynamics of the density matrix $\rho_{AF}$ are determined by the equation:

(12) $$\frac{\partial \rho_{AF}}{\partial t}+\frac{i}{\hbar }\left[H_{AF},\rho_{AF}\right]=0\;.$$

It should be complemented by the terms describing the recombination and relaxation processes, including the relaxation between the bright and dark exciton states, as well as the spin relaxation of the bright and dark exciton pseudospins. In the case of cw excitation, the steady-state bright and dark average pseudospins can be found from:

(13) $$\begin{array}{c} S_{A}\times \Omega_{A}=\frac{S_{A}^{0}-S_{A}}{\tau_{A}}-\frac{S_{A}-S_{A}^{\mathrm{eq}}}{\tau_{sA}}, \end{array}$$

(14) $$\begin{array}{c} S_{F}\times \Omega_{F}=\frac{S_{F}^{0}-S_{F}}{\tau_{F}}-\frac{S_{F}-S_{F}^{\mathrm{eq}}}{\tau_{sF}}\;. \end{array}$$

Here, the left-hand terms in both equations describe the rotation of the pseudospins in the effective magnetic fields directed along $\Omega_{A}=\left(\Omega_{X},0,\Omega_{Z}\right)$ and $\Omega_{F}=\left(\Omega_{FX},0,\Omega_{FZ}\right)$ for the bright and dark exciton, respectively, with the effective Larmor frequencies $\Omega_{A}=\sqrt{\Omega_{X}^{2}+\Omega_{Z}^{2}}$ and $\Omega_{F}=\sqrt{\Omega_{FX}^{2}+\Omega_{FZ}^{2}}$. These fields include both the external magnetic field directed along *Z* and the anisotropic field directed along *X* in the pseudospin space.

The right-hand terms describe the balance between the pseudospin generation and recombination processes controlled by the bright and dark exciton lifetimes, $\tau_{A}$ and $\tau_{F}$, and the pseudospin relaxation processes controlled by the bright and dark exciton spin relaxation times, $\tau_{sA}$ and $\tau_{sF}$. The relaxation processes tend to bring the average pseudospins to the thermodynamic equilibrium pseudospin values $S_{A(F)}^{\mathrm{eq}}$, directed along the effective fields and given by:

(15) $$S_{A(F)}^{\mathrm{eq}}=\frac{\Omega_{A(F)}}{2\Omega_{A(F)}}\tanh\frac{\hbar \Omega_{A(F)}}{2k_{B}T}\;.$$

Hereafter, we will restrict the theoretical consideration to the low-temperature regime in which the bright exciton pseudospin $S_{A}^{0}$ is created only by the exciting light as described by Equation (9). The dark exciton pseudospin $S_{F}^{0}$ can be generated only via the relaxation of the polarized population from the bright exciton state, so that $S_{F\gamma }^{0}={\hat{G}}_{\gamma \beta }S_{A\beta }$, where ${\hat{G}}_{\gamma \beta }$ (γ,β=X,Y,Z) is the second-rank polarization transfer tensor. Along with the assumption that m=±2 states inherit the circular polarization selection rules from the m=±1 states, the circular polarization is expected to be fully transferred from the bright to the dark exciton with ${\hat{G}}_{Z\beta }=\delta_{Z\beta }$, where $\delta_{\gamma \beta }$ is the Kronecker symbol. The respective relaxation processes between the bright and dark exciton |±1〉, |±2〉 sublevels are shown in Figure S3c. In turn, the transfer of the linear polarization with ${\hat{G}}_{X\beta }=\pm \delta_{X\beta }$ is possible only in the case of a strong in-plane anisotropy of the internal exchange field that couples the bright and the dark exciton (which also provides the linear polarization of the dark exciton emission discussed above) [44]. The respective relaxation processes between linearly polarized sublevels of the bright and dark excitons are shown in Figure S3b.

We will seek solutions for Equations (13) and (14) in the form of $S_{A(F)}=S_{A1(F1)}+S_{A2(F2)}$, where $S_{A1(F1)}$ are directed parallel and $S_{A2(F2)}$ are transverse to the effective fields $\Omega_{A(F)}$, respectively. It is easy to show that with restrictions $\Omega_{F}T_{F}\gg 1$ and $\Omega_{A}T_{A}\gg 1$, allowing many pseudospin rotations around the effective field during the spin lifetime, the transverse to the effective fields solutions vanish and the steady-state pseudospin is always directed along the effective field [24,35]. Here, the bright (dark) exciton pseudospin lifetimes $T_{A(F)}$ are defined as $1/T_{A(F)}=1/\tau_{A(F)}+1/\tau_{sA(sF)}$. The parallel solutions have both non-equilibrium and equilibrium contributions and can be written as follows:

(16) $$\begin{array}{c} S_{A1}=\frac{T_{A}}{\tau_{A}}\frac{\left(S_{A}^{0}\Omega_{A}\right)\Omega_{A}}{\Omega_{A}^{2}}+\frac{T_{A}}{\tau_{sA}}S_{A}^{\mathrm{eq}}\;, \end{array}$$

(17) $$\begin{array}{c} S_{F1}=\frac{T_{F}}{\tau_{F}}\frac{\left(S_{F}^{0}\Omega_{F}\right)\Omega_{F}}{\Omega_{F}^{2}}+\frac{T_{F}}{\tau_{sF}}S_{F}^{\mathrm{eq}}\;. \end{array}$$

One can see that the applied magnetic field in the Faraday geometry converts the linearly polarized dipoles |X〉,|Y〉 into circular components |+1〉, |−1〉 (Figure 5b). This restores the circular polarization of the PL when $\Omega_{Z}T_{A}\gg 1$. However, the $S_{Y}$ component is always vanishing in the considered geometry and the $S_{Z}$ component is vanishing in the zero magnetic field. For this reason, the $\Omega_{A}T_{A}\gg 1$ restriction does not allow one to describe the effects of the rotation of the linear polarization plane, observed in the experiment and expected to arise from the bright exciton recombination, while only circular polarization of the dark exciton recombination is expected. Therefore, we further consider the steady-state solutions of Equations (13) and (14) for $S_{A}$, assuming arbitrary relations between $1/T_{A}$ and $\Omega_{A}$. Then, the transverse to the effective field contribution to the bright exciton average pseudospin can be written as

(18) $$\begin{array}{c} S_{A2}=\frac{T_{A}}{\tau_{A}}\frac{T_{A}}{1+\Omega_{A}^{2}T_{A}^{2}}\left(\Omega_{A}\times S_{A}^{0}+\frac{\Omega_{A}\times \left[S_{A}^{0}\times \Omega_{A}\right]}{\Omega_{A}^{2}T_{A}}\right)\;. \end{array}$$

The average pseudospin steady-state components $S_{A,\gamma }$ (γ=X,Y,Z) are found as $S_{A,\gamma }=S_{A1,\gamma }+S_{A2,\gamma }$. Their explicit form in a certain magnetic field in the Faraday geometry is given in Supplementary Materials, Section S3, Equation (S12).

### 3.3. PL Polarization from an Individual NPL and an Ensemble of Randomly Oriented NPLs

Combining the steady-state solutions for the bright exciton pseudospin components, i.e., Equations (S10) and (S11) with Equations (9) and (11), one can find the bright exciton contributions to the circular, $P_{c}^{c}\left(\alpha \right)$, and linear, $P_{l}^{l}\left(\alpha \right)$, $P_{l^{\prime }}^{l}\left(\alpha \right)$, polarizations that are proportional to the circular, $P_{0}^{c}$, or the linear, $P_{0}^{l}$, polarizations of the incident light in an individual NPL with the angle α between the axis *X* and the laboratory axis *x* (Figure 5a). The bright exciton contributions are weighted by the factor $A=I_{A}/\left(I_{A}+I_{F}\right)$. The circular polarization of the PL does not depend on α, and the optical orientation effect coming from the $S_{AZ}$ component generated by $P_{0}^{c}$ is the same in all NPLs. In contrast, the linear polarization of the PL strongly depends on α. The resulting expressions for the bright exciton contributions to the $P_{l}^{l}\left(\alpha \right)$ and the $P_{l^{\prime }}^{l}\left(\alpha \right)$ coming from the initial $P_{0}^{l}$ excitation are presented in the Supplementary Materials, Section S3 (see Equation (S13)).

Note that the fixed angle α describes an ensemble of in-plane-oriented horizontal NPLs. The Lorentzian fit used for the estimation of the amplitudes and HWHMs of the optical alignment contours (Figure 2c) corresponds to such an oriented ensemble with α=0. However, in our samples, the NPLs are randomly in-plane-oriented (Figure 1b). For the ensemble averaging, we consider randomly in-plane-oriented NPLs lying on the substrate, having the same anisotropic splitting $\Omega_{X(FX)}$ and the same exciton *g*-factors, $g_{A(F)}$. Since the total PL intensity does not depend on the in-plane orientation of the NPL, only the averaging of the polarizations coming from each NPL over the angle α is needed. After averaging with a uniform distribution f=1/2π over the angle α, we obtain the results for the three bright exciton contributions to the Stokes parameters in the laboratory frame:

(19) $$\begin{array}{c} P_{lA}^{l}=AP_{0}^{l}\frac{T_{A}}{2\tau_{A}}\frac{\left(2+\Omega_{X}^{2}T_{A}^{2}\right)}{\left(1+\Omega_{A}^{2}T_{A}^{2}\right)},\\ P_{l^{\prime }A}^{l}=AP_{0}^{l}\frac{T_{A}}{\tau_{A}}\frac{T_{A}\Omega_{Z}}{\left(1+\Omega_{A}^{2}T_{A}^{2}\right)},\\ P_{cA}^{c}=AP_{0}^{c}\frac{T_{A}}{\tau_{A}}\frac{\left(1+\Omega_{Z}^{2}T_{A}^{2}\right)}{\left(1+\Omega_{A}^{2}T_{A}^{2}\right)}. \end{array}$$

The conversion of circular to linear polarizations, and *vice versa*, is vanishing after averaging.

In addition, there is a dark exciton contribution to the optical orientation effect proportional to $F=I_{F}/\left(I_{A}+I_{F}\right)$ in the nonzero magnetic field coming from the $S_{FZ}$ pseudospin component. In the limiting case $\Omega_{F}T_{F}\gg 1$, it is found to be

(20) $$\begin{array}{c} P_{cF}^{c}=FP_{0}^{c}\frac{T_{F}}{\tau_{F}}\frac{T_{A}}{\tau_{A}}\frac{\left(1+\Omega_{Z}^{2}T_{A}^{2}\right)}{\left(1+\Omega_{A}^{2}T_{A}^{2}\right)}\frac{\Omega_{FZ}^{2}}{\Omega_{F}^{2}}\;. \end{array}$$

The contribution from the dark exciton, $P_{lF}^{l}$, to the linear polarization, is generally not expected in the case of the random orientation of the internal magnetic field coupling the bright and dark excitons. However, possible evidence of such a contribution is discussed in the next section in relation to the modeling of the experimental data.

For any polarization of the incident light, there are also thermodynamic equilibrium contributions to the linear and circular polarizations in the individual NPLs. The linear polarization vanishes by the angular averaging over the ensemble of horizontally lying NPLs. The circular polarization does not depend on the angle α and comes from both the bright, $S_{AZ}^{\mathrm{eq}}$, and dark, $S_{FZ}^{\mathrm{eq}}$, exciton pseudospins. They change the sign with the reversal of the magnetic field direction and can be distinguished by the optical orientation effects in the experiment.

To summarize, in this section, we provide the theoretical blocks needed to describe the optical alignment and optical orientation effects in the Faraday magnetic field for an ensemble of colloidal NPLs. Below is the analysis of the experimental data using the developed theory in order to quantitatively determine the exciton parameter ranges.

## 4. Modeling of Experimental Data

### 4.1. Analysis of Magnetic Field Polarization Dependence

Here, we analyze the experimental magnetic-field dependence of the three polarization parameters, $P_{l}^{l}$, $P_{l^{\prime }}^{l}$, and $P_{c}^{c}$, shown in Figure 2b,d for the low-temperature regime. The resulting model curves are placed within the same Figures by solid lines. We start from the contributions coming from the bright excitons resulting in the broad contours in all three polarization dependences for both chosen detection energies. We use the theoretical expressions of Equation (19) with the additional parameter $P_{l}^{\mathrm{const}}$ added to $P_{l}^{l}$ to take into account the constant linear polarization from the vertically oriented NPLs.

For the sake of convenience of the data analysis, we introduce the dimensionless variables $X_{A}=\Omega_{X}T_{A}$ and $Z_{A}=\Omega_{Z}\left(B_{1}\right)T_{A}$, where $B_{1}=1$ T. We neglect the possible dependence of the spin relaxation time and, thus, of the spin lifetime $T_{A}$ on the magnetic field, so that $\Omega_{Z}\left(B\right)T_{A}=Z_{A}B/B_{1}$ for any magnetic field *B*.

We remind the reader that the observed circular and linear polarizations can be affected by the depolarization factor coming from the recombination of excitons in vertically oriented NPLs as well as by the initial loss of polarization in the case of nonresonant excitation. Both effects are taken into account by allowing the initial polarizations $P_{0}^{l},P_{0}^{c}\leq 1$. At low temperatures, the factor $A=\Gamma_{A}\tau_{A}\left(\Gamma_{A}+\gamma_{0}\right)/\left(\Gamma_{A}+\eta \gamma_{0}\right)$, where $\eta =\eta_{F}/\eta_{A}$. To minimize the number of parameters, we set η=1 and introduce the variable $A_{0}=AP_{0}^{l}T_{A}/\tau_{A}=P_{0}^{l}\Gamma_{A}T_{A}$ and write the bright exciton contributions to the detected polarizations as follows:

(21) $$\begin{array}{ccc} P_{lA}^{l}\left(B\right) & = & \frac{A_{0}}{2}\left(\frac{2+X_{A}^{2}}{1+X_{A}^{2}+Z_{A}^{2}{\left(B/B_{1}\right)}^{2}}\right)+P_{l}^{\mathrm{const}}\;,\\ P_{l^{\prime }A}^{l}\left(B\right) & = & A_{0}\frac{Z_{A}B/B_{1}}{\left(1+X_{A}^{2}+Z_{A}^{2}{\left(B/B_{1}\right)}^{2}\right)},\\ P_{cA}^{c}\left(B\right) & = & \frac{P_{0}^{c}}{P_{0}^{l}}A_{0}\frac{1+Z_{A}^{2}{\left(B/B_{1}\right)}^{2}}{1+X_{A}^{2}+Z_{A}^{2}{\left(B/B_{1}\right)}^{2}}\;. \end{array}$$

The sets of bright exciton parameters, allowing a good description of the polarization dependences on the magnetic field in the Faraday geometry for the two detection energies, are presented in Table 1. The evaluation procedure is described in more detail in the Supplementary Materials, Section S5.

The pairs of the parameters $X_{A}$ and $Z_{A}$, presented in Table 1, comprise, in turn, an infinite number of particular values of the bright exciton dynamics and fine structure parameters $T_{A}$, $\Omega_{X}=X_{A}/T_{A}$, and $g_{A}=\hbar Z_{A}/\mu_{B}B_{1}T_{A}$. It is our aim now to estimate the possible ranges of these parameters. The upper limit for the bright exciton spin lifetime $T_{A}$ is given by the bright exciton lifetime $\tau_{A}=0.9$ ns, determined from the PL decay data (Figure 4a). The lower limit for close-to-resonance detection can be estimated as $T_{A}=A_{0}/\Gamma_{A}$. The bright exciton recombination rate of $\Gamma_{A}=0.8$ ns${}^{-1}$ and $P_{0}^{l}=1$ results in $0.1\;\mathrm{ns}\leq T_{A}\leq 0.9\;\mathrm{ns}$. We use the same range for $T_{A}$ in the case of nonresonant detection so that the decrease in the optical alignment amplitude $A_{0}$ is related to the decrease in $P_{0}^{l}<1$. Such an approach allows us to conclude that the exciton parameters do not vary much across the PL line. However, at this stage, we cannot attempt their precise determination, and we estimate only the possible ranges of their values.

Thus, for the spin lifetime range $0.1\;\mathrm{ns}\leq T_{A}\leq 0.9\;\mathrm{ns}$, we obtain the following intervals for the anisotropic splitting of states and the *g*-factor of the bright exciton: 0.9 μeV $\leq \hbar \Omega_{X}\leq 7.6$ μeV, and $0.011\leq g_{A}\leq 0.095$ (see Table S1 in Supplementary Materials, Section S5) and 1.3 μeV $\leq \hbar \Omega_{X}\leq 10.8$ μeV, and $0.019\leq g_{A}\leq 0.154$ (see Table S2 in Supplementary Materials, Section S5). The corresponding range of the spin relaxation rates is much wider. For a more accurate determination of the bright exciton spin relaxation rate and the respective anisotropic splitting, time-resolved studies of the optical alignment and orientation effects are needed, which are beyond the scope of this paper.

### 4.2. Dark Exciton Contribution to the Ensemble Polarization

As has been already described above, the presence of dark exciton radiative recombination results in the renormalization of the PL polarization (Equation (10)). Let us analyze now the direct contribution of the polarization of the dark exciton PL. It adds at least to the effect of optical orientation according to Equation (20) and is weighted by the factor $F=1-A=\gamma_{0}\tau_{A}$ at low temperatures. The effect vanishes at zero magnetic field in the considered limit $\Omega_{XF}T_{F}\gg 1$, however, it may contribute at finite external magnetic field. To estimate this contribution by using Equation (20), we need to know two additional parameters: the dark exciton spin lifetime $T_{F}$ and the anisotropic splitting $\hbar \Omega_{FX}$. We remind that the dark exciton lifetime $\tau_{F}=250$ ns was evaluated from the PL decay (Figure 4). We can estimate the dark exciton *g*-factor using the relations for the bright and dark exciton *g*-factors:

(22) $$\begin{array}{c} g_{A}=-g_{e}-3g_{h},\\ g_{F}=g_{e}-3g_{h}, \end{array}$$

where $g_{e(h)}$ is the electron (hole) *g*-factor. The value of $g_{e}=1.67$ for the electron is obtained by spin-flip Raman scattering spectroscopy (Figure 3d). However, the hole *g*-factor is unknown for the studied NPLs. In Ref. [31], regarding CdSe NPLs with thick shells, the value of $g_{h}=-0.4$ was obtained in low magnetic fields. Using the range for the bright exciton *g*-factor $0.011\leq g_{A}\leq 0.095$ (see Table S1 in Supplementary Materials, Section S5), we obtain $g_{h}=-\left(g_{e}+g_{A}\right)/3\approx -0.6$, comparable with the value from Ref. [31] and thereby determine the range for the dark exciton *g*-factor $g_{F}=2g_{e}+g_{A}$, as $3.35\leq g_{F}\leq 3.44$.

Such a large value of the dark exciton *g*-factor allows us to suggest that the narrow part of the optical alignment $P_{l}^{l}\left(B\right)$ dependence, which is present in Figure 2b and is absent in Figure 2d, is related to the dark exciton. As discussed in the theory, it is not expected (and is even surprising) that its amplitude is nonzero. The reason for this may be the anisotropy of the internal magnetic field that couples the bright and dark excitons [44].

The HWHM of the narrow contour is obtained in the field of $B_{F1/2}∼0.1\;T$. In the dark exciton contribution, $P_{lF}^{l}$, given in the Supplementary Materials, Equation (S18), the multiplier associated with the bright exciton in such a small field is still close to that at zero field. Therefore, the condition for the HWHM of the narrow contour can be obtained as follows:

(23) $$\frac{P_{lF}^{l}\left(B_{F1/2}\right)}{P_{lF}^{l}\left(0\right)}=\frac{\left(1+\Omega_{FX}^{2}T_{F}^{2}\right)}{\left(1+\Omega_{F}^{2}T_{F}^{2}\right)}=\frac{1}{2}\;.$$

In the limiting case $\Omega_{FX}T_{F}\gg 1$, this condition results in $\Omega_{FZ}\left(B_{F1/2}\right)=\Omega_{FX}$. We will see below that this condition is well-satisfied for the dark exciton. With the determined dark exciton *g*-factor, it allows us to estimate the range 19.4μeV $\leq \hbar \Omega_{FX}\leq 19.9\;\mu$eV.

The dark exciton contribution to the amplitude of the optical alignment effect is given by:

(24) $$P_{lF}^{l}\left(0\right)=A_{0}\frac{T_{F}\gamma_{0}}{2\Gamma_{A}\tau_{F}}\;,$$

enabling us to estimate the dark exciton spin lifetime $T_{F}=245$ ns to be very close to the lifetime $\tau_{F}=233$ ns, resulting in the much longer spin relaxation time $\tau_{sF}={\left(1/T_{F}-1/\tau_{F}\right)}^{-1}\approx 3.5$ μs. Thus, for the determined value of $T_{F}$, we obtain $\Omega_{FX}T_{F}∼7\times 10^{3}$, justifying the used approximation $\Omega_{FX}T_{F}\gg 1$. The set of dark exciton parameters, allowing a good description of the narrow part of the optical alignment dependence on the magnetic field in the Faraday geometry, is shown in Table 1.

We used the determined parameters of the dark exciton to plot the dark exciton contribution to the optical orientation effect, according to Equation (20), both for resonant and nonresonant detection (see Figure 2b,d and Figure S4 in the Supplementary Materials, Section S5).

## 5. Discussion and Conclusions

We determined the parameters of the exciton fine structure (see Table 1) with good accuracy by analyzing the PL polarization from an ensemble of CdSe/CdS NPLs under polarized cw excitation. For the modeling of the data and determination of the parameters, we used the simplest model without considering such effects as the anisotropy of the spin relaxation time and its dependence on the external magnetic field. The detailed theory, taking into account the anisotropy of the spin relaxation time, can be found in Ref. [44]. We provide the final expressions of this theory in Supplementary Materials, Section S3. We use it to fit the experimental data; the results are presented in the Supplementary Materials, Section S5 (Figure S4 and Tables S3 and S4).

For the simplest model, taking into account the spin relaxation time anisotropy, the largest remaining uncertainty is the anisotropic splitting between the spin sublevels of the bright exciton and its spin relaxation time. For better access to these parameters, as well as for the analysis of the magnetic field dependence of the spin relaxation time, in the future, we plan to perform time-resolved studies of the optical alignment and optical orientation effects in NPLs.

The main contribution to all three effects (optical alignment, rotation of the linear polarization plane, and optical orientation) originates from the bright exciton that was excited resonantly by polarized light. The presence of radiative recombination from the dark exciton states populated after relaxation from the bright exciton brings new features to the polarization of the PL. First of all, it acts as a depolarization factor for the recombination from the bright exciton. Second, there is always the dark exciton contribution to the optical orientation effect in the nonzero Faraday magnetic field. We suggest that there is a transfer of the optical alignment from the bright to the dark exciton taking place only under strongly resonant conditions (when the difference between the detection and excitation energies is about 5 meV). Studies of the microscopic origin of such a transfer and its resonant character are beyond the scope of this paper. However, such a suggestion allows us to estimate the spin lifetime and the anisotropic splitting of the dark exciton states in zero magnetic field. The latter turns out to be larger than that of the bright exciton states. Importantly, these splittings, $\hbar \Omega_{X}$ and $\hbar \Omega_{XF}$, have a different nature. The splitting between the states of the bright exciton is driven by the anisotropy of the NPLs in the plane, which indeed is not large for the studied NPLs. For the dark exciton, the cubic $J_{\alpha }^{3}\sigma_{\alpha }$ exchange term also leads to the splitting of its states. It is the small anisotropic splitting of the bright exciton $\hbar \Omega_{X}≲10\;\mu$eV in the studied NPLs that allowed us to observe the rotation of the linear polarization plane in the magnetic field in the Faraday geometry, as well as the optical orientation in zero magnetic field.

It is important to note that the effects of the conversion from linear to circular polarization and *vice versa* vanish upon averaging over the randomly oriented NPL ensemble. However, the estimated parameters allow us to model the effects in the individual NPL and an oriented ensemble, as shown in Figure 5c,d. In addition to the random orientation of the NPLs in the ensemble, the exciton parameters in the NPLs, such as the anisotropic exciton splittings $\hbar \Omega_{X}$ and $\hbar \Omega_{XF}$, can be characterized by some dispersion in the ensemble. To account for it, additional averaging with a distribution function can be carried out, as conducted, for example, in Ref. [37], for the ensemble of perovskite nanocrystals.

To conclude, we presented the first observation of exciton optical alignment and optical orientation in an ensemble of core/shell CdSe/CdS colloidal NPLs. We developed a theoretical model, describing the dependences of these effects on the magnetic field in the Faraday geometry. We determined all main parameters of the exciton fine structure and dynamic processes in the studied NPLs. The presence of the CdS shell in the studied CdSe/CdS NPLs resulted in a decrease in the electron–hole exchange interaction and a corresponding decrease in the energy splitting between the bright and dark exciton states, as well as of the relaxation rate between them, in comparison to the CdSe NPLs without the shell. We conclude that the observation of the optical alignment and optical orientation effects in core/shell CdSe/CdS NPLs is possible due to the strong contribution of the bright exciton to the PL intensity, even at low temperatures.

## 6. Methods

**Sample fabrication.** A set of CdSe/CdS core/shell NPLs with different shell thicknesses is studied here. The fabrication procedure is described in Refs. [45,46]. The parameters of all studied samples can be found in Table 1 of Ref. [33]. The NPLs are passivated with oleic acid and stored in a mixed solvent consisting of 40% heptane and 60% decane. All NPL samples were grown from the same CdSe core with an average lateral dimension of (13.7±0.2)×(10.8±0.2) nm${}^{2}$ and a thickness of 1.2 nm (i.e., 4 monolayers). The CdSe/CdS NPLs have a total thickness of 3.8±0.5 nm (very thin shell), 4.6±0.6 nm (thin), 7.4±1.0 nm (medium shell), 11.6±1.6 nm (thick shell), 19.1±1.6 nm (very thick shell), including the thicknesses of the CdSe core and CdS shells on both sides. For performing low-temperature experiments, the NPLs in solvent are drop-cast on a Si substrate and dried. Typically, drop-cast core/shell NPLs have two preferable orientations on the substrate, as schematically shown in Figure 1, i.e., vertical NPLs with the c-axis parallel to the substrate plane, and horizontal NPLs with the c-axis perpendicular to the substrate plane. Although the effects in all samples are similar, the results reported here focus on the data for the NPLs with a medium-shell thickness (sample number MP170214A) of 3.1 nm on each side of the core.

**Continuous wave excitation experiment.** For polarized PL spectroscopy and Raman scattering (RS) spectroscopy, the sample is placed in the variable temperature insert (1.5–300 K) of a helium bath cryostat with a superconducting solenoid (up to 5 T). The magnetic field is applied either parallel to the light wave vector (Faraday geometry) or perpendicular to it (Voigt geometry). For the excitations of PL and RS, a DCM dye laser is used. The laser power density focused on the sample does not exceed 5 W/cm${}^{2}$. The laser spot on the sample has a diameter of approximately 300 μm. The PL and RS are measured in backscattering geometry and analyzed by a Jobin Yvon U1000 double monochromator equipped with a cooled GaAs photomultiplier and conventional photon-counting electronics. The linear and circular polarizations of the PL are measured using a photoelastic modulator (PEM) in the detection path. The PEM modulates the circular polarization of light between $\sigma^{+}$ and $\sigma^{-}$ at the frequency of 42 kHz, synchronized with the detector. Together with a linear polarizer and a λ/4 plate, the PEM allows one to measure the PL polarization $P_{l}$, $P_{l^{\prime }}$, and $P_{c}$, as described in the main text.

**Polarized PL spectroscopy.** The polarization of light is characterized by the three Stokes parameters. Two of them characterize the linear polarization degree along the *x*, *y* axes and $x^{\prime }$, $y^{\prime }$ axes rotated by 45${}^{\circ }$ degrees around the *z*-axis ($P_{l}$ and $P_{l^{\prime }}$, respectively). The third one characterizes the circular polarization degree ($P_{c}$). Thus, the Stokes parameters are defined as follows:

$$P_{l}=\frac{I_{x}-I_{y}}{I_{x}+I_{y}}\;,\;\;P_{l^{\prime }}=\frac{I_{x^{\prime }}-I_{y^{\prime }}}{I_{x^{\prime }}+I_{y^{\prime }}}\;,\;\;P_{c}=\frac{I_{+}-I_{-}}{I_{+}+I_{-}}\;.$$

Here, $I_{x(y)}$ is the intensity of the horizontally (vertically) linearly polarized component, $I_{x^{\prime }\left(y^{\prime }\right)}$ is the intensity of the 45${}^{\circ }$ ($-45^{\circ }$) polarized component, and $I_{+(-)}$ is the intensity of the $\sigma^{+}$ ($\sigma^{-}$) circularly polarized component of light. Note that we keep the definition of the circular polarization sign with respect to the same direction of the *z*-axis (Figure 1) for both the exciting and emitted lights.

In general, one can perform nine measurements, taking into account the combination of the three options for the polarization of the incident and detected light. We denote each measurement as $P_{\alpha }^{\beta }$, which combines the α-polarized detection $P_{\alpha }$ and the β-polarized excitation $P_{0}^{\beta }$ with $\alpha ,\beta =c,l,l^{\prime }$. For the ensemble of NPLs, two preferred orientations of the NPL anisotropic c-axis with respect to the substrate are expected: vertically oriented NPLs (Figure 1a) with the c-axis lying in the substrate plane and horizontally oriented NPLs (Figure 1b) with the c-axis perpendicular to the substrate [12,31]. For these samples, due to the random in-plane orientation of the NPLs on the substrate, the optical alignment is independent of the specific linear polarization of the exciting light ($P_{l}^{l}∼P_{l^{\prime }}^{l^{\prime }}$), which is also true for the effect of the rotation of the linear polarization plane ($P_{l^{\prime }}^{l}∼P_{l}^{l^{\prime }}$). For the same reason, all the effects associated with the conversion of the initial linear polarization to circular polarization and *vice versa* are absent, as evidenced by the vanishing experimental dependence on the magnetic field $P_{c}^{l}\left(B\right)=P_{l}^{c}\left(B\right)=P_{c}^{l^{\prime }}\left(B\right)=P_{l^{\prime }}^{c}\left(B\right)=0$. Therefore, to describe polarization-dependent effects, it is sufficient to investigate three nontrivial results: the effect of optical orientation, measured as $P_{c}^{c}$, the effect of optical alignment, measured as $P_{l}^{l}$, and the effect of rotation of the linear polarization plane, measured as $P_{l^{\prime }}^{l}$. We study these three effects in magnetic fields of up to 5 T, applied in the Faraday geometry with B‖k, where k is the wave vector of the emitted light being opposite to the wave vector of the exciting light directed perpendicular to the substrate. While $P_{l}^{l}$ and $P_{c}^{c}$ are nonzero in the absence of a magnetic field, and are even with respect to the sign of the magnetic field *B*, $P_{l^{\prime }}^{l}$ manifests itself only in the nonzero magnetic field in the Faraday geometry and is odd with respect to the magnetic field. The measured degree of the circular polarization contains two contributions: the optical orientation and the thermodynamic contribution caused by the difference in populations of the energy-split spin states in the external magnetic field. The latter is odd with respect to the magnetic field and does not depend on the specific circular polarization of the exciting light. Thus, to exclude this contribution, we measure the degree of the circular polarization with the circular excitation of both signs ($\sigma^{+}$ and $\sigma^{-}$) and calculate the optical orientation as $P_{c}^{c}=\left(P_{\sigma +}^{c}-P_{\sigma -}^{c}\right)/2$.

**Time-resolved experiments.** The sample is placed into a bath cryostat with a variable temperature insert (1.5–300 K) and a superconducting solenoid (up to 6 T). As photoexcitation sources, we use two semiconductor pulsed lasers with photon energies of 2.380 eV and 1.958 eV, a pulse duration of 50 ps, and a repetition rate ranging from 0.5 MHz to 5 MHz. The average power of photoexcitation was held at 1 mW/cm${}^{2}$. The PL is spectrally resolved by a double spectrometer with 900 g/mm gratings in the dispersion subtraction regime. A part of the PL band with a width of less than 0.5 nm is detected by a photomultiplier tube used for photon counting, and measured with time resolution with a conventional time-correlated single photon-counting setup (instrumental response about 100 ps).

## Figures and Tables

**Table 1:** Sets of the bright and dark exciton parameters for the two detection energies, $E_{\det1}=1.955$ eV and $E_{\det2}=1.943$ eV, allowing for an accurate description of the polarization dependences on the magnetic field in the Faraday geometry.

	$X_{A}$	$Z_{A}$	$A_{0}$	$P_{0}^{c}/P_{0}^{l}$	$P_{l}^{\mathrm{const}}$
$E_{\det1}$	1.1	0.8	0.08	0.71	0.110
$E_{\det2}$	1.6	1.4	0.06	0.45	0.058
	$\hbar \Omega_{FX}$μeV	$g_{F}$	$T_{F}$	$\tau_{sF}$	$\Omega_{FX}T_{F}$
$E_{\det1}$	19.65	3.4	233 ns	3.5 μs	$7\times 10^{3}$

## Data Availability

The data presented in this study are available upon request from the corresponding authors.

## Supplementary Materials

The following supporting information can be downloaded at https://www.mdpi.com/article/10.3390/nano13172402/s1, S1. Additional experimental data are on PL spectra under different conditions, PL and Raman scattering in NPLs of different thicknesses, temperature-dependent TRPL; S2. Exciton kinetic equations; S3. Bright exciton pseudospin components in the Faraday magnetic field; S4. Dark exciton contribution to the PL polarization; S5. Details on the data analysis; S6. NPL characterization: TEM images and room temperature PL spectra; Table S5. Notations used in the paper.
