# Supplementary material for: Optical Alignment and Optical Orientation of Excitons in CdSe/CdS Colloidal Nanoplatelets
Source: Nanomaterials (Basel). 2023 Aug 24;13(17):2402. doi: 10.3390/nano13172402 (PMC10489814; doi:10.3390/nano13172402)
Supplement: Supplementary file 1 [file nanomaterials-13-02402-s001.zip › nanomaterials-2552552-supplementary.pdf]

Supplementary Materials

Optical Alignment and Optical Orientation of Excitons in CdSe/CdS Colloidal Nanoplatelets

Olga O. Smirnova[1,*], Ina V. Kalitukha[1,2,*], Anna V. Rodina[1,*], Grigorii S. Dimitriev[1], Victor F. Sapega[1], Olga S. Ken[1], Vladimir L. Korenev[1], Nikolai V. Kozyrev[1], Sergey V. Nekrasov[1], Yuri G. Kusrayev[1], Dmitri R. Yakovlev[1,2,*], Benoit Dubertret[3], and Manfred Bayer[2]

[1]*Ioffe Institute, Russian Academy of Sciences, 194021 St. Petersburg, Russia*
[2]*Experimentelle Physik 2, Technische Universität Dortmund, 44221 Dortmund, Germany*
[3]*Laboratoire de Physique et d'étude des Matériaux, ESPCI, CNRS, 75231 Paris, France*
*Correspondence: smirnova.olga@mail.ioffe.ru (O.O.S.); kalitukha@gmail.com (I.V.K.); anna.rodina@mail.ioffe.ru (A.V.R.); dmitri.yakovlev@tu-dortmund.de (D.R.Y.)

S1. Additional experimental data

In this section we present additional experimental data measured with cw (Figure S1) and pulsed (Figure S2) excitation.

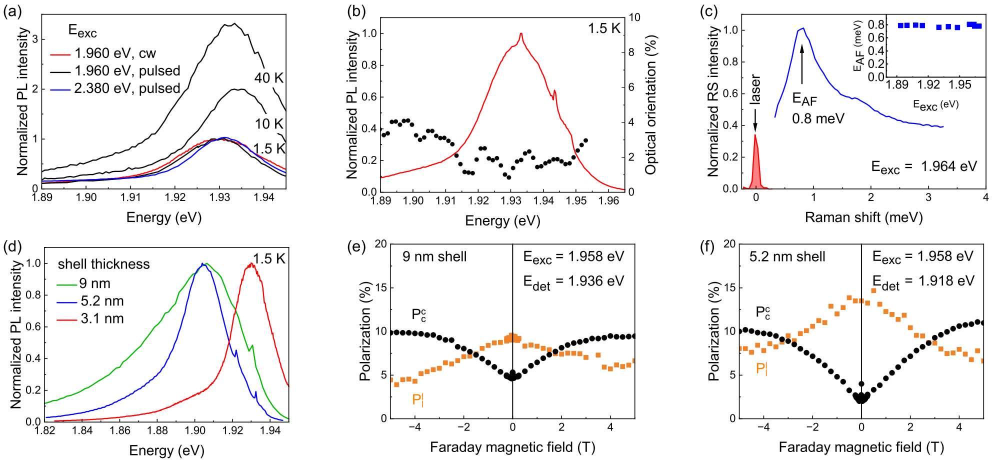

Figure S1. PL and RS spectroscopy of colloidal CdSe/CdS NPLs. (a) Normalized PL spectra at $T = 1.5$ K forr resonant cw (red) and pulsed (black) excitation and nonresonant pulsed (blue) excitation. PL spectra (black, pulsed resonant excitation) at different temperatures, normalized in way to conserve the ratio between PL intensities. (b) Normalized PL spectrum (red) and optical orientation spectrum (black circles) at $T = 1.5$ K for resonant cw excitation. (c) Raman scattering spectrum for excitation energy $E_{\text{exc}} = 1.964$ eV at zero magnetic field with $T = 1.5$ K. Laser is shown by the red filled curve. Inset: Dependence of the bright–dark exciton energy splitting E_{AF} on the excitation energy. (d) Normalized PL spectra of NPLs with different shell thickness at $T = 1.5$ K for resonant cw excitation. Faraday magnetic field dependences of the linear polarization P_l^l (orange squares) and the optical orientation P_c^c (black circles) for NPLs with 9 nm (e) and 5.2 nm (f) shell thickness.

S2. Exciton kinetic equations

For square nanoplatelets (NPLs) in the absence of an external magnetic field, the system can be considered within the framework of a three-level model consisting of the ground state $|G\rangle$ and the states of the bright $|A\rangle$ and dark $|F\rangle$ exciton (Figure S3a). The presence of the splitting between the sublevels of the bright and the dark excitons $\hbar\Omega_X$ and $\hbar\Omega_{FX}$, respectively (Figure S3b), requires that all four levels are taken into account explicitly.

The presence of relaxation between the $|X\rangle$ ($|Y\rangle$) and $|FX\rangle$ ($|FY\rangle$) states, as shown by the lilac and crimson arrows in Figure S3b, allows one to obtain the dark exciton

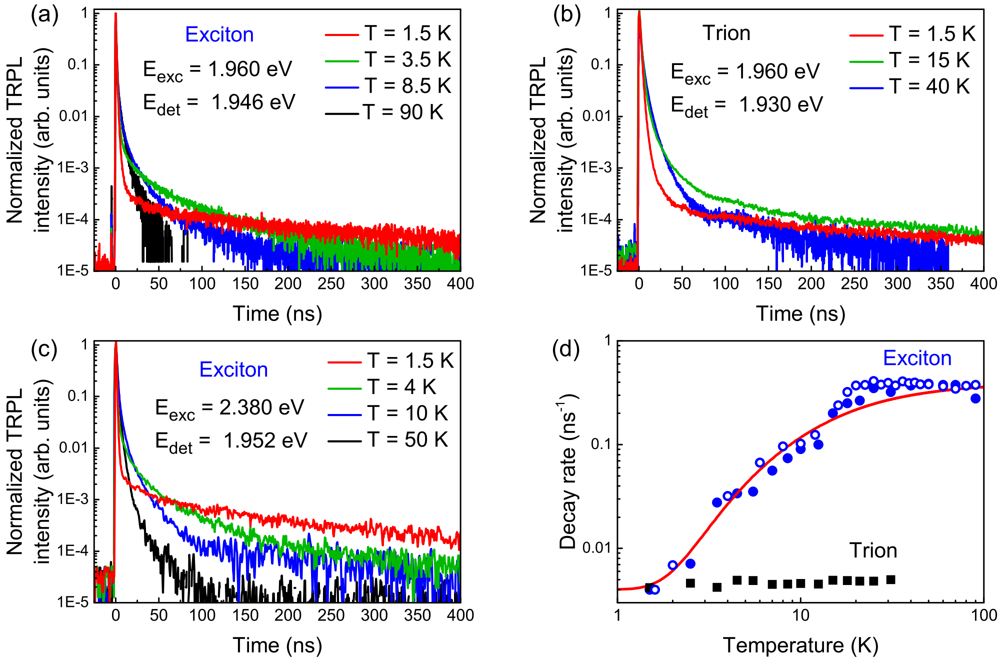

Figure S2. Time-resolved PL spectroscopy of the colloidal CdSe/CdS NPLs in a wide temperature range. (a) PL dynamics for resonant excitation detected at the exciton. (b) PL dynamics for resonant excitation detected on the trion. (c) PL dynamics for nonresonant excitation detected on the exciton. (d) Experimental dependence of the decay rate Γ_L on the temperature for resonant (blue circles) and nonresonant (blue open circles) excitation of the exciton and for resonant excitation of the trion (black squares). Theoretical curve is given by the red line.

contribution to the optical alignment effect within the model. In absence of the magnetic field, the energy relaxation is associated with a transition between the linear components $|X\rangle$, $|Y\rangle$ and similarly composed dark exciton states $|FX\rangle$, $|FY\rangle$ and takes a characteristic time τ_{sA} (τ_{s1} in case of an anisotropy). In a strong magnetic field in the Faraday geometry, when the anisotropic splitting becomes insignificant, the energy relaxation during time τ_{sA} (τ_{s1}) occurs between the $|+1\rangle$ ($|-1\rangle$) and $|+2\rangle$ ($|-2\rangle$) states, as shown by the lilac and crimson arrows in Figure S3c.

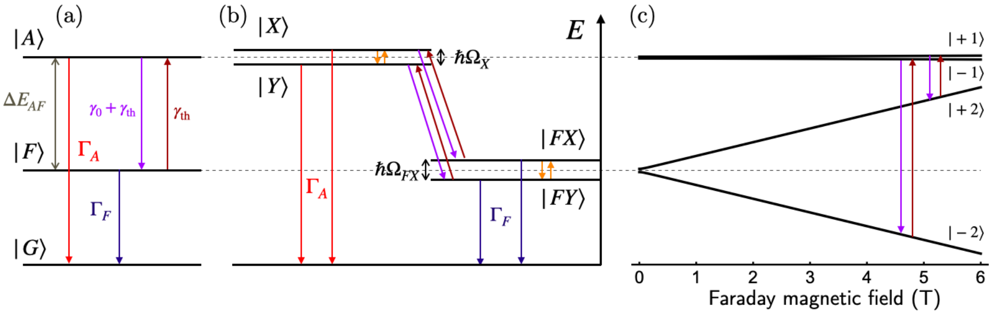

Figure S3. (a) Scheme of the lower energy levels of the bright $|A\rangle(\pm 1)$ and dark $|F\rangle(\pm 2)$ excitons split by ΔE_{AF}, (b) the considered transitions between the states of the bright and dark excitons with characteristic rates in the absence of an external magnetic field. These transitions between the bright and dark exciton states allow the linear components of the pseudospin to be preserved. (c) Splitting of the states in the Faraday magnetic field using the parameters obtained during the analysis.

The system of rate equations describing the transfer of exciton populations N_A and N_F between the bright and dark exciton levels, taking into account the recombination and the relaxation between them, can be written in the following matrix form:

$$\frac{\partial}{\partial t}\begin{pmatrix} N_A \\ N_F \end{pmatrix} = A\begin{pmatrix} N_A \\ N_F \end{pmatrix} + \begin{pmatrix} G_A \\ G_F \end{pmatrix}, \tag{S1}$$

$$A = \begin{pmatrix} -(\Gamma_A + \gamma_0 + \gamma_{\text{th}}) & \gamma_{\text{th}} \\ \gamma_0 + \gamma_{\text{th}} & -(\Gamma_F + \gamma_{\text{th}}) \end{pmatrix} = \begin{pmatrix} -\frac{1}{\tau_A} & \gamma_{\text{th}} \\ \gamma_0 + \gamma_{\text{th}} & -\frac{1}{\tau_F} \end{pmatrix},$$

where $\Gamma_{A(F)}$ is the radiation recombination rate of the bright (dark) exciton, γ_0 is the relaxation rate from the bright state to the dark one at zero temperature, γ_{th} is the thermally activated phonon-assisted relaxation $\gamma_{\text{th}} = \gamma_0 N_B$, where $N_B(\Delta E_{AF})$ is the Bose–Einstein phonon occupation $N_B(E_{\text{ph}}) = 1/(\exp(E_{\text{ph}}/k_B T) - 1)$, k_B is the Boltzmann constant, E_{ph} is the phonon energy. The resulting lifetimes of the bright and dark excitons are given by:

$$\tau_A^{-1} = \Gamma_A + \gamma_0 + \gamma_{\text{th}}, \quad \tau_F^{-1} = \Gamma_F + \gamma_{\text{th}}. \tag{S2}$$

Exciton generation is determined by $G_{A(F)}$ $(G_A + G_F = 1)$. We consider two ways of optical excitation: continuous wave (cw) and pulsed excitation. In the first case, we are interested in the stationary solutions with $G_A(t), G_F(t) = \text{const}$:

$$N_A = \frac{\tau_A(G_A + (1 - \Gamma_F\tau_F)G_F)}{\Gamma_F\tau_F + \Gamma_A\tau_A(1 - \Gamma_F\tau_F)},$$
$$N_F = \frac{\tau_F((1 - \Gamma_A\tau_A)G_A + G_F)}{\Gamma_F\tau_F + \Gamma_A\tau_A(1 - \Gamma_F\tau_F)}. \tag{S3}$$

For quasi-resonant excitation we assume that only the bright exciton with much larger oscillator strength is excited ($G_A = 1, G_F = 0$). At low temperature $\Gamma_F\tau_F = 1$. Therefore, $N_A = \tau_A G_A$ and $N_F = \tau_F(1 - \Gamma_A\tau_A)G_A = \tau_F\tau_A\gamma_0 G_A$.

In the second regime the states are excited by a laser pulse at the initial moment of time. For resonant excitation we similarly assume that only the bright exciton is excited $[N_A(0) = 1, N_F(0) = 0]$. In the case of nonresonant excitation after supposedly fast relaxation from the excited level higher in energy we consider $N_A(0) = N_F(0) = 0.5$. The dynamics of the total photoluminescence (PL) intensity from the system can be represented as follows:

$$I(t) = \Gamma_A^{\text{r}} N_A(t) + \Gamma_F^{\text{r}} N_F(t) = B_1 \exp(-\Gamma_S t) + B_2 \exp(-\Gamma_L t)\,, \tag{S4}$$

where $\Gamma^{\text{r}}_{A(F)} = \eta_{A(F)}\Gamma_{A(F)}$, $\eta_{A(F)}$ is the bright (dark) exciton radiative efficiency. Γ_S and Γ_L are the eigenvalues of the matrix A (Equations (S1)) and the constants B_1, B_2 can be found from the initial conditions. In the absence of relaxation from the lower levels, when $\gamma_{\text{th}} = 0$, which is satisfied at zero temperature, the eigenvalues are equal to the inverse of the lifetimes τ_A^{-1} and τ_F^{-1}.

The analysis of the PL dynamics makes it possible to determine some of the involved parameters, namely, the recombination rates of the bright, Γ_A, and dark, Γ_F, excitons, the energy splitting between these levels, ΔE_{AF}, and the relaxation rate at zero temperature γ_0. The expression for the fast (short), Γ_S, and slow (long), Γ_L, PL decay rates in terms of the three-level model can be written as follows [1]:

$$\Gamma_S = \frac{1}{2}\left(\Gamma_A + \Gamma_F + \gamma_0 \coth\left(\frac{\Delta E_{AF}}{2k_B T}\right) + \sqrt{(\gamma_0 + \Gamma_A - \Gamma_F)^2 + \gamma_0^2 \sinh^{-2}\left(\frac{\Delta E_{AF}}{2k_B T}\right)}\right), \tag{S5}$$

$$\Gamma_L = \frac{1}{2}\left(\Gamma_A + \Gamma_F + \gamma_0 \coth\left(\frac{\Delta E_{AF}}{2k_B T}\right) - \sqrt{(\gamma_0 + \Gamma_A - \Gamma_F)^2 + \gamma_0^2 \sinh^{-2}\left(\frac{\Delta E_{AF}}{2k_B T}\right)}\right). \tag{S6}$$

At low temperature, $\Gamma_L = \Gamma_F$, and at saturation with temperature, $\Gamma_L = \frac{\Gamma_A + \Gamma_F}{2}$. The parameters γ_0 and ΔE_{AF} determine the temperature dependence. The fast component at low temperature (Figure 4a) allows one to fix the sum $\Gamma_A + \gamma_0 = 1.11$ ns^{-1}.

A magnetic field in the Voigt geometry mixes the bright and dark exciton states, causing additional activation of the radiative recombination of the lower energy states [2]:

$$\Gamma_F = \Gamma_F(B=0) + \left(\frac{g_e \mu_B B}{\Delta E_{AF}}\right)^2 \Gamma_A. \tag{S7}$$

The experimentally measured dependences of the asymptotic rate on temperature and on the magnitude of the magnetic field in the Voigt geometry are shown in Figures 4c and 4d, respectively. The data can be consistently described with the theoretical dependences using the following parameter values $\Gamma_A = 0.8$ ns^{-1}, $\Gamma_F = 0.004$ ns^{-1}, $\gamma_0 = 0.3$ ns^{-1}, $\Delta E_{AF} = 0.8$ meV. This set of parameters also allows us to describe the temperature dependence of Γ_L in the case of nonresonant excitation at the energy $E_{\mathrm{exc}} = 2.380$ eV (Figure S2d).

S3. Bright exciton pseudospin components in the Faraday magnetic field

Here, we provide more details about the bright exciton pseudospin $\mathbf{S}_A$. In the case of cw excitation, the steady-state bright exciton average pseudospins can be found from the equation:

$$\mathbf{S}_A \times \mathbf{\Omega}_A = \frac{\mathbf{S}_A^0 - \mathbf{S}_A}{\tau_A} - \frac{\mathbf{S}_A - \mathbf{S}_A^{\mathrm{eq}}}{\tau_{sA}}. \tag{S8}$$

The left hand term describes the rotation of a pseudospin in the effective magnetic field directed along $\mathbf{\Omega}_A = (\Omega_X, 0, \Omega_Z)$ with the effective Larmor frequency $\Omega_A = \sqrt{\Omega_X^2 + \Omega_Z^2}$. This field includes both the external magnetic field directed along Z and the anisotropic field directed along X in the pseudospin space.

The right hand terms describe the balance between the pseudospin generation and recombination processes controlled by the bright exciton lifetime τ_A and the pseudospin relaxation processes controlled by the exciton spin relaxation time τ_{sA}. The relaxation processes tend to bring the average pseudospin to the thermodynamical equilibrium pseudospin $\mathbf{S}_A^{\mathrm{eq}}$, directed along the effective field, given by:

$$\mathbf{S}_A^{\mathrm{eq}} = \frac{\mathbf{\Omega}_A}{2\Omega_A} \tanh \frac{\hbar \Omega_A}{2 k_{\mathrm{B}} T}.$$

In general, to take into account the spin relaxation time anisotropy, we represent $\mathbf{S}_A = \mathbf{S}_{A1} + \mathbf{S}_{A2}$, where the $\mathbf{S}_{A1}$ are directed parallel and the $\mathbf{S}_{A2}$ transverse to the effective field $\mathbf{\Omega}_A$, respectively, and rewrite Equation (S8) as

$$\mathbf{S}_A \times \mathbf{\Omega}_A = \frac{\mathbf{S}_A^0 - \mathbf{S}_A}{\tau_A} - \frac{\mathbf{S}_{A1} - \mathbf{S}_A^{\mathrm{eq}}}{\tau_{s1}} - \frac{\mathbf{S}_{A2} - \mathbf{S}_A^{\mathrm{eq}}}{\tau_{s2}} \tag{S9}$$

where τ_{s1} and τ_{s2} are the longitudinal and transverse spin relaxation times. The solutions can be found as

$$\mathbf{S}_{A1} = \frac{T_1}{\tau_A} \frac{(\mathbf{S}_A^0 \mathbf{\Omega}_A)\mathbf{\Omega}_A}{\Omega_A^2} + \frac{T_1}{\tau_{sA}} \mathbf{S}_A^{\mathrm{eq}}, \tag{S10}$$

$$\mathbf{S}_{A2} = \frac{T_2}{\tau_A} \frac{T_2}{1 + \Omega_A^2 T_2^2} \left(\mathbf{\Omega}_A \times \mathbf{S}_A^0 + \frac{\mathbf{\Omega}_A \times [\mathbf{S}_A^0 \times \mathbf{\Omega}_A]}{\Omega_A^2 T_2} \right). \tag{S11}$$

Here, the exciton pseudospin lifetimes $T_{1(2)}$ are defined as $1/T_{1(2)} = 1/\tau_A + 1/\tau_{s1(s2)}$.

The average pseudospin steady-state components $S_{A,\gamma}$ ($\gamma = X, Y, Z$) can be found as $S_{A,\gamma} = S_{A1,\gamma} + S_{A2,\gamma}$. Their explicit form in a certain magnetic field applied in the Faraday geometry in the case $T_1 = T_2 = T_A$ can be written as

$$\begin{aligned} S_{AX} &= \frac{T_A(S^0_{AX}(1+\Omega_X^2 T_A^2) - \Omega_Z T_A S^0_{AY} + \Omega_Z\Omega_X T_A^2 S^0_{AZ})}{\tau_A(1+\Omega_A^2 T_A^2)}, \\ S_{AY} &= \frac{T_A(S^0_{AY} + \Omega_Z T_A S^0_{AX} - \Omega_X T_A S^0_{AZ})}{\tau_A(1+\Omega_A^2 T_A^2)}, \\ S_{AZ} &= \frac{T_A(S^0_{AZ}(1+\Omega_Z^2 T_A^2) + \Omega_X T_A S^0_{AY} + \Omega_X\Omega_Z T_A^2 S^0_{AX})}{\tau_A(1+\Omega_A^2 T_A^2)}. \end{aligned} \tag{S12}$$

Without simplification, in the case when $T_1 \neq T_2$, the expressions for the pseudospin components take the form

$$\begin{aligned} S_{AX} &= \frac{S^0_{AX}(T_2\Omega_Z^2 + T_1\Omega_X^2(1+T_2^2\Omega_A^2)) - S^0_{AY}T_2^2\Omega_Z\Omega_A^2 + S^0_Z\Omega_X\Omega_Z(T_1(1+T_2^2\Omega_A^2) - T_2)}{\tau_A\Omega_A^2(1+\Omega_A^2 T_2^2)}, \\ S_{AY} &= \frac{T_2(S^0_{AY} + S^0_{AX}T_2\Omega_Z - S^0_Z T_2\Omega_X)}{\tau_A(1+\Omega_A^2 T_2^2)}, \\ S_{AZ} &= \frac{S^0_Z(T_2\Omega_X^2 + T_1\Omega_Z^2(1+T_2^2\Omega_A^2)) + S^0_{AY}T_2^2\Omega_X\Omega_A^2 + S^0_{AX}\Omega_X\Omega_Z(T_1(1+T_2^2\Omega_A^2) - T_2)}{\tau_A\Omega_A^2(1+\Omega_A^2 T_2^2)}. \end{aligned} \tag{S13}$$

The obtained averaged pseudospin components allow us to write the linear and circular polarizations in the individual NPLs and in the ensemble

$$\begin{aligned} P^l_{lA}(\alpha) &= \mathcal{A}\frac{T_A P^l_0}{\tau_A(1+\Omega_A^2 T_A^2)}\Big(1+\Omega_X^2 T_A^2\cos^2(2\alpha)\Big), \\ P^l_{l'A}(\alpha) &= \mathcal{A}\frac{T_A P^l_0}{\tau_A(1+\Omega_A^2 T_A^2)}\left(\Omega_Z T_A - \Omega_X^2 T_A^2\frac{\sin(4\alpha)}{2}\right), \\ P^l_{cA}(\alpha) &= \mathcal{A}\frac{T_A P^l_0}{\tau_A(1+\Omega_A^2 T_A^2)}\left(\Omega_Z\Omega_X T_A^2\cos(2\alpha) + \Omega_X T_A\sin(2\alpha)\right), \end{aligned} \tag{S14}$$

where $\mathcal{A} = I_A/(I_A + I_F)$ is the relative intensity of the bright exciton, $I_{A,F} = \Gamma^{\mathrm{r}}_{A,F}N_{A,F}$. The results for the ensemble are written in Equations (19) in the main text:

$$P^l_{lA} = \mathcal{A}P^l_0\frac{T_A}{2\tau_A}\frac{(2+\Omega_X^2 T_A^2)}{(1+\Omega_A^2 T_A^2)},\quad P^l_{l'A} = \mathcal{A}P^l_0\frac{T_A}{\tau_A}\frac{T_A\Omega_Z}{(1+\Omega_A^2 T_A^2)},\quad P^c_{cA} = \mathcal{A}P^c_0\frac{T_A}{\tau_A}\frac{(1+\Omega_Z^2 T_A^2)}{(1+\Omega_A^2 T_A^2)}. \tag{S15}$$

We can also write the resulting polarizations for the anisotropic case:

$$P_{lA}^{l}(\alpha) = \frac{\mathcal{A}P_0^l}{\tau_A(1+\Omega_A^2T_2^2)}\left(\frac{T_1+T_2}{2}\cos^2(2\alpha) + T_2\sin^2(2\alpha) + T_1T_2^2\Omega_X^2\cos^2(2\alpha)\right)+$$
$$+(T_1-T_2)\frac{P_0^l(\Omega_X^2-\Omega_Z^2)}{2\tau_A\Omega_A^2(1+\Omega_A^2T_2^2)}\cos^2(2\alpha),$$
$$P_{l'A}^{l}(\alpha) = \mathcal{A}\frac{P_0^lT_2}{\tau_A(1+\Omega_A^2T_2^2)}\left(\Omega_ZT_2 - T_1T_2\Omega_X^2\frac{\sin(4\alpha)}{2}\right)-$$
$$-(T_1-T_2)\frac{P_0^l\Omega_X^2}{\tau_A\Omega_A^2(1+\Omega_A^2T_2^2)}\frac{\sin(4\alpha)}{2}, \tag{S16}$$
$$P_{cA}^{l}(\alpha) = \mathcal{A}\frac{P_0^lT_2}{\tau_A(1+\Omega_A^2T_2^2)}(\Omega_X\Omega_ZT_1T_2\cos(2\alpha) + T_2\Omega_X\sin(2\alpha))+$$
$$+(T_1-T_2)\frac{P_0^l\Omega_X\Omega_Z}{\tau_A\Omega_A^2(1+2\Omega_A^2T_2^2)}\cos(2\alpha).$$

The answers for the effects in the ensemble with an anisotropic pseudospin lifetime are given by:

$$P_{lA}^{l} = \mathcal{A}\frac{P_0^l}{2\tau_A}\left(\frac{T_2(2+\Omega_X^2T_2^2)}{(1+\Omega_A^2T_2^2)} + (T_1-T_2)\frac{\Omega_X^2}{\Omega_A^2}\right),$$
$$P_{l'A}^{l} = \mathcal{A}\frac{P_0^l}{\tau_A}\frac{T_2^2\Omega_Z}{1+\Omega_A^2T_2^2}, \tag{S17}$$
$$P_{cA}^{c} = \mathcal{A}\frac{P_0^c}{\tau_A}\left(\frac{T_2(1+\Omega_Z^2T_2^2)}{(1+\Omega_A^2T_2^2)} + (T_1-T_2)\frac{\Omega_Z^2}{\Omega_A^2}\right).$$

S4. Dark exciton contribution to the photoluminescense polarization

The main contributions from the dark exciton to the measured optical alignment and optical orientation effects are the following:

$$P_{lF}^{l} = \mathcal{F}\frac{T_FP_l^0}{2\tau_F}\frac{(1+\Omega_{FX}^2T_F^2)}{(1+\Omega_F^2T_F^2)}\frac{T_A(1+\Omega_X^2T_A^2)}{\tau_A(1+\Omega_A^2T_A^2)}, \tag{S18}$$

$$P_{cF}^{c} = \mathcal{F}P_0^c\frac{T_F}{\tau_F}\frac{T_A}{\tau_A}\frac{(1+\Omega_{FZ}^2T_F^2)}{(1+\Omega_F^2T_F^2)}\frac{(1+\Omega_Z^2T_A^2)}{(1+\Omega_A^2T_A^2)}. \tag{S19}$$

In the case of the relaxation time anisotropy taken into account for the bright exciton, the contributions of the dark exciton also change their form:

$$P_{lF}^{l} = \mathcal{F}\frac{T_FP_0^l}{2\tau_F}\frac{(1+\Omega_{FX}^2T_F^2)}{(1+\Omega_F^2T_F^2)}\cdot\frac{\Omega_Z^2T_2 + T_1\Omega_X^2(1+\Omega_A^2T_2^2)}{\tau_A\Omega_A^2(1+\Omega_A^2T_2^2)}, \tag{S20}$$
$$P_{cF}^{c} = \mathcal{F}\frac{T_FP_0^c}{\tau_F}\frac{(1+\Omega_{FZ}^2T_F^2)}{(1+\Omega_F^2T_F^2)}\cdot\frac{\Omega_X^2T_2 + \Omega_Z^2T_1(1+\Omega_A^2T_2^2)}{\tau_A\Omega_A^2(1+\Omega_A^2T_2^2)}. \tag{S21}$$

S5. Details on the data analysis

For a simplified model, the analysis of the experimental data is reduced to two conditions that allow us to introduce the parameter fixings $X_A = \Omega_XT_A$ and $Z_A = \Omega_ZT_A(B_1)$, where $B_1 = 1$ T. One condition is imposed on the ratio of the difference of the optical

alignment effect as compared with its value in zero magnetic field (helping to exclude a constant contribution) to the rotation of the linear polarization plane:

$$\frac{P_l^l(B=0)-P_l^l(B)}{P_{l'}^l(B)}=\frac{(2+X_A^2)Z_A}{(1+X_A^2)}\frac{B}{B_1}=K_1B. \tag{S22}$$

The experimental data cannot be described by a linear dependence on the magnetic field, which indicates the inaccuracy of the simple model used. To analyze the data, we focused on the magnetic field range of $3-4$ T. For the data obtained the detection at the energy $E_{\text{det}}=1.955$ eV we obtain $K_1=0.60$. When detected at $E_{\text{det}}=1.943$ eV we obtain $K_1=0.86$.

Another condition is imposed on the ratio of the recovering optical orientation in a certain magnetic field to its value in zero magnetic field. Unity is subtracted for convenience:

$$\frac{P_c^c(B)}{P_c^c(B=0)}-1=\frac{X_A^2Z_A^2}{1+X_A^2}\left(\frac{B}{B_1}\right)^2=K_2B^2. \tag{S23}$$

For this condition, the quadratic magnetic field dependence describes the experimental data quite well in magnetic fields ~ 0.5 T. For the data at the energy $E_{\text{det}}=1.955$ eV we obtain $K_2=0.40$. When detected at $E_{\text{det}}=1.943$ eV we obtain $K_2=1.33$.

The constants K_1 and K_2 allow us to fix the parameters $X_A=1.1$ and $Z_A=0.8$ for $E_{\text{det}}=1.955$ eV and $X_A=1.6$ and $Z_A=1.4$ for $E_{\text{det}}=1.943$ eV.

Then we can reconstruct the rotation amplitude (by fixing the product $\Gamma_A P_0^l T_A$) and the constant contribution to the linear polarization P_l^{const}, and determine the ratio P_0^c/P_0^l. For the obtained parameters, given that the pseudospin lifetime 0.1 ns $\leq T_A \leq$ 0.9 ns, we obtain possible ranges of the exciton parameters: spin relaxation time τ_{sA}, g-factor of the bright exciton g_A, anisotropic splitting between linear components of the bright exciton $\hbar\Omega_X$. We also obtain the initial polarization P_0^l. These parameters are presented in Tables S1 and S2 for the detection energies $E_{\text{det}}=1.955$ eV and $E_{\text{det}}=1.943$ eV, respectively. The theoretical modeling for the experimental data with the obtained parameters is shown in Figure S4a for $E_{\text{det}}=1.955$ eV and in Figure S4c for $E_{\text{det}}=1.943$ eV.

Table S1. Sets of the bright exciton parameters in the case of detection at $E_{\text{det}}=1.955$ eV, allowing description of the broad part of the polarization dependences on the magnetic field in the Faraday geometry.

$X_A=\Omega_X T_A$	$Z_A=\Omega_Z T_A$	$\mathcal{A}_0$	P_0^c/P_0^l	P_l^{const}
1.1	0.8	0.08	0.71	0.110
T_A, ns	τ_{sA}, ns	P_0^l	g_A	$\hbar\Omega_X$, μeV
0.1	0.11	1.00	0.095	7.6
0.2	0.26	0.52	0.048	3.8
0.3	0.45	0.35	0.032	2.5
0.4	0.72	0.26	0.024	1.9
0.5	1.13	0.21	0.019	1.5
0.6	1.80	0.17	0.016	1.3
0.7	3.15	0.15	0.014	1.1
0.8	7.20	0.13	0.012	1.0
0.85	15.30	0.12	0.011	0.9

In Figure S4 the dark exciton contributions to the effects are also taken into account. Circular polarization is added at both detection energies. In the alignment, the contribution appears only when detected at the energy $E_{\text{det}}=1.955$ eV in the form of a narrow contour. From this contour, we have further defined the parameters of the dark exciton. Given a fixed value of g_A and the known g_e (Figure 3d), we have determined the eange $3.35 \leq g_F \leq 3.44$ (Equations (22)). The magnetic-field dependence of the contribution gives a splitting

Table S2. Sets of the bright exciton parameters in the case of detection at $E_{\rm det} = 1.943$ eV allowing description of the broad part of the polarization dependences on the magnetic field in the Faraday geometry.

$X_A = \Omega_X T_A$	$Z_A = \Omega_Z T_A$	$\mathcal{A}_0$	P_0^c/P_0^l	$P_l^{\rm const}$
1.6	1.4	0.06	0.45	0.058

T_A, ns	τ_{sA}, ns	P_0^l	g_A	$\hbar\Omega_X$, μeV
0.1	0.11	0.72	0.154	10.8
0.2	0.26	0.36	0.077	5.4
0.3	0.45	0.24	0.051	3.6
0.4	0.72	0.18	0.038	2.7
0.5	1.13	0.15	0.031	2.2
0.6	1.80	0.12	0.026	1.8
0.7	3.15	0.10	0.022	1.5
0.8	7.20	0.09	0.019	1.4
0.85	15.30	0.09	0.018	1.3

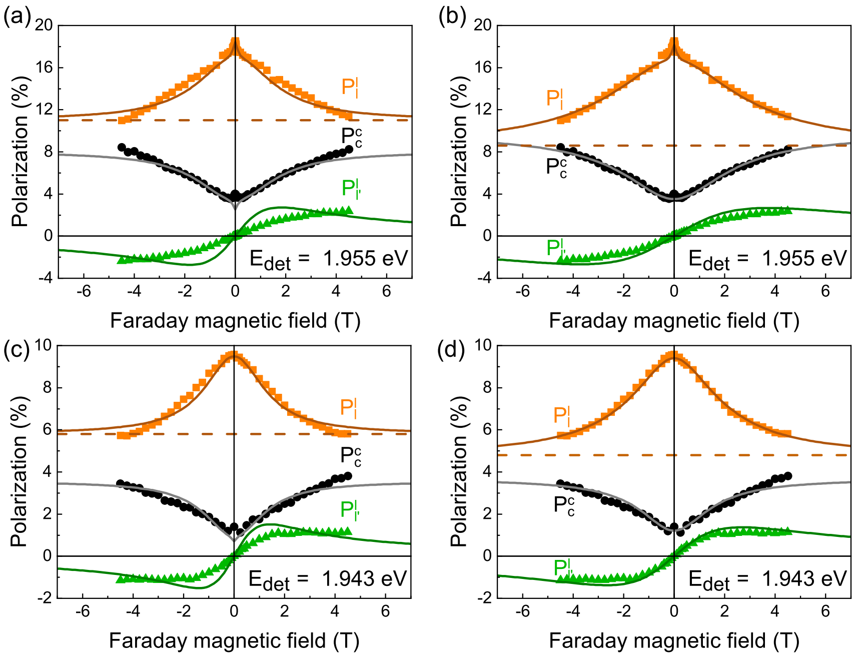

Figure S4. Polarized PL of CdSe/CdS NPLs for resonant excitation at $E_{\rm exc} = 1.960$ eV. Experimental data (symbols) and theoretical calculations (solid lines of corresponding colors) of the optical alignment (orange squares), the rotation of the linear polarization plane (green triangles) and the optical orientation (black circles) measured in the Faraday geometry at (a), (b) $E_{\rm det} = 1.955$ eV and at (c), (d) $E_{\rm det} = 1.943$ eV. The theoretical curves are plotted (a), (c) using the simple model without taking into account the relaxation time anisotropy and (b), (d) with its incorporation. The constant for the optical alignment effect is (a) 11.0%, (b) 8.6%, (c) 5.8%, (d) 4.8%.

19.4 μeV $\leq \hbar\Omega_{FX} \leq$ 19.9 μeV. The amplitude of this contribution corresponds to the lifetime $T_F = 233$ ns.

To analyze the experimental data in the framework of the model taking into account the relaxation time anisotropy, we also introduce the parameters $X_A = \Omega_X T_1$ and $Z_A = \Omega_Z T_1$ and the parameter to characterize the anisotropy $t_{12} = T_1/T_2$. Here, Z_A is also fixed in a certain field B. In this analysis, we take a field close to the HWHM of the broad contour of the optical alignment effect $B = 3$ T for $E_{\rm det} = 1.955$ eV and $B = 2$ T for $E_{\rm det} = 1.943$ eV. By using the experimental data, three conditions are imposed:

$$\begin{aligned}
\frac{P^l_{lA}(B)}{P^l_{lA}(B=0)} &= \frac{(1+X_A^2)(2Z_A^2+X_A^2(1+t_{12}(1+X_A^2+Z_A^2)))}{(1+t_{12}(1+X_A^2))(X_A^2+Z_A^2)(1+X_A^2+Z_A^2)}\,, \\
\frac{P^l_{l'A}(B)}{P^l_{lA}(B)} &= \frac{2Z_A(X_A^2+Z_A^2)}{2Z_A^2+X_A^2(1+t_{12}(1+X_A^2+Z_A^2))}\,, \\
\frac{P^c_{cA}(B=0)}{P^c_{cA}(B)} &= \frac{(X_A^2+Z_A^2)(1+X_A^2+Z_A^2)}{(1+X_A^2)(X_A^2+t_{12}Z_A^2(1+X_A^2+Z_A^2))}\,.
\end{aligned} \tag{S24}$$

For the data obtained at the detection energy $E_{\text{det}} = 1.955$ eV the parameters are $X_A = 1.3$, $Z_A = 1.8$ and $t_{12} = 1.8$. For the second data set with $E_{\text{det}} = 1.943$ eV, $X_A = 1.4$, $Z_A = 1.9$ and $t_{12} = 2.1$. The exciton parameter sets are presented in Tables S3 and S4. The dark exciton parameters are: $T_F = 80$ ns, $\hbar\Omega_{FX} \approx 11.6$ μeV. The resulting theoretical curves are shown as solid lines in Figures S4b and S4d.

Table S3. Sets of the bright exciton parameters obtained by means of a model accounting for relaxation time anisotropy in the case of detection at $E_{\text{det}} = 1.955$ eV allowing description of the polarization dependences on the magnetic field in the Faraday geometry.

$t_{12} = T_1/T_2$	$X_A = \Omega_X T_1$	$Z_A = \Omega_Z T_1$	$\mathcal{A}_0 = \Gamma_A P_l^0 T_1$	P_0^c/P_0^l	P_l^{const}
1.8	1.3	1.8	0.12	0.81	0.086
T_1, ns	τ_{s1}, ns	τ_{s2}, ns	P_0^l	g_A	$\hbar\Omega_X$, μeV
0.2	0.26	0.13	0.91	0.034	4.2
0.3	0.45	0.20	0.61	0.023	2.8
0.4	0.72	0.29	0.46	0.017	2.1
0.5	1.13	0.40	0.37	0.014	1.7
0.6	1.80	0.53	0.30	0.011	1.4
0.7	3.15	0.68	0.26	0.010	1.2
0.8	7.20	0.88	0.23	0.009	1.1
0.85	15.30	0.99	0.22	0.008	1.0

Table S4. Sets of the bright exciton parameters obtained by means of a model accounting for relaxation time anisotropy in the case of detection at $E_{\text{det}} = 1.943$ eV allowing description of the polarization dependences on the magnetic field in the Faraday geometry.

$t_{12} = T_1/T_2$	$X_A = \Omega_X T_1$	$Z_A = \Omega_Z T_1$	$\mathcal{A}_0 = \Gamma_A P_l^0 T_1$	P_0^c/P_0^l	P_l^{const}
2.1	1.4	1.9	0.07	0.53	0.048
T_1, ns	τ_{s1}, ns	τ_{s2}, ns	P_0^l	g_A	$\hbar\Omega_X$, ¯eV
0.2	0.26	0.11	0.43	0.053	4.6
0.3	0.45	0.17	0.29	0.035	3.0
0.4	0.72	0.24	0.22	0.027	2.3
0.5	1.13	0.32	0.17	0.021	1.8
0.6	1.8	0.42	0.14	0.018	1.5
0.7	3.15	0.53	0.12	0.015	1.3
0.8	7.20	0.66	0.11	0.013	1.1
0.85	15.30	0.74	0.10	0.013	1.1

S6. Characterization of nanoplatelets

Characterization of the studied core/shell CdSe/CdS NPLs includes TEM images (Figure S5) and room temperature PL spectra (Figure S6).

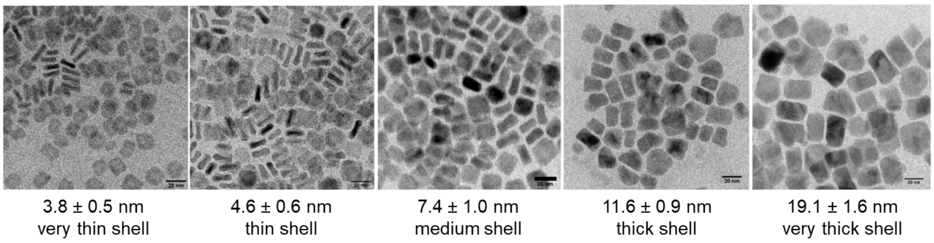

Figure S5. TEM images of the core/shell CdSe/CdS NPLs. Below each TEM image the complete thickness of the shown NPLs is indicated. TEM image of NPLs, discussed in the main text, is marked as “medium shell”.

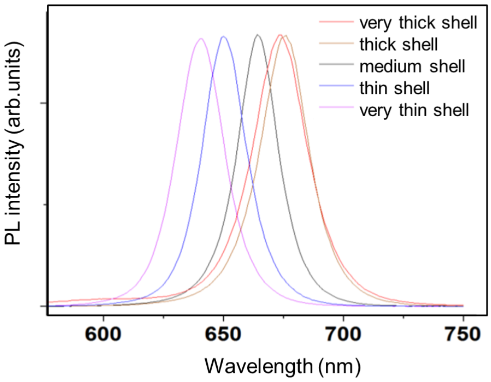

Figure S6. Room temperature PL spectra of core/shell CdSe/CdS NPLs. The sample labels correspond to Figure S5.

References

[1] Shornikova, E. V.; Biadala, L.; Yakovlev, D. R.; Sapega, V. F.; Kusrayev, Yu. G.; Mitioglu, A. A.; Ballottin, M. V.; Christianen, P. C. M.; Belykh, V. V.; Kochiev, M. V.; Sibeldin, N. N.; Golovatenko, A. A.; Rodina, A. V.; Gippius, N. A.; Kuntzmann, A.; Jiang, Y.; Nasilowski, M.; Dubertret, B.; Bayer, M. Addressing the exciton fine structure in colloidal nanocrystals: the case of CdSe nanoplatelets. *Nanoscale* **2018**, *10*, 646–656. https://doi.org/10.1039/C7NR07206F

[2] Rodina, A. V.; Efros, Al. L. Radiative recombination from dark excitons in nanocrystals: Activation mechanisms and polarization properties. *Phys. Rev.* B **2016**, *93*, 15542. https://link.aps.org/doi/10.1103/PhysRevB.93.155427

Table S5. Notations used in the paper

Notation	Definition
P_l^l -	degree of the PL linear polarization in the laboratory frame under linearly polarized laser excitation
P_l^{const} -	linear polarization related to structural/orientational anisotropy
$P_{l'}^l$ -	degree of the PL linear polarization in rotated by 45° laboratory frame under linearly-polarized excitation
P_c^c -	degree of the PL circular polarization under circularly polarized excitation
P_0^l -	degree of exciting light linear polarization
P_0^c -	degree of exciting light circular polarization
$P_{L,L',C}$ -	polarization of light emitted by excitons from a single horizontally oriented NPL
ΔE_{AF} -	bright–dark exciton energy splitting
E_{exc} -	excitation energy
E_{det} -	detection energy
g_e -	electron g-factor
g_h -	hole g-factor
g_A -	bright exciton g-factor
g_F -	dark exciton g-factor
Γ_A -	recombination rate of the bright exciton
Γ_F -	recombination rate of the dark exciton
Γ_A^{r} -	radiative recombination rate of the bright exciton
Γ_F^{r} -	radiative recombination rate of the dark exciton
η_A -	bright exciton radiative recombination efficiency
η_F -	bright and dark exciton radiative recombination efficiency
N_B -	phonon occupation
γ_{th} -	phonon-assisted relaxation rate, $\gamma_{\text{th}} = \gamma_0 N_B$
γ_0 -	relaxation rate from the bright to the dark exciton state at zero temperature
τ_A -	bright exciton lifetime
τ_F -	dark exciton lifetime
Γ_S -	decay rate of the short component of the PL intensity
Γ_L -	decay rate of the long component of the PL intensity
N_A -	bright exciton state population
N_F -	dark exciton state population
I_A -	intensity of the bright exciton, $I_A = \Gamma_A^{\text{r}} N_A$
I_F -	intensity of the dark exciton, $I_F = \Gamma_F^{\text{r}} N_F$
I -	total intensity of the exciton photoluminescence, $I = I_A + I_F$
$\mathcal{A}$ -	bright exciton relative intensity, $\mathcal{A} = I_A/(I_A + I_F)$
$\mathcal{F}$ -	dark exciton relative intensity, $\mathcal{F} = I_F/(I_A + I_F)$
$\mathbf{S}_A$ -	bright exciton averaged pseudospin
$\mathbf{S}_F$ -	dark exciton averaged pseudospin
$\mathbf{S}_A^{\text{eq}}$ -	thermodynamic equilibrium pseudospin of bright exciton
$\mathbf{S}_F^{\text{eq}}$ -	thermodynamic equilibrium pseudospin of dark exciton
$\hbar\Omega_X$ -	energy splitting between the $\lvert X\rangle$ and $\lvert Y\rangle$ bright exciton states in absence of an external magnetic field
$\hbar\Omega_{FX}$ -	energy splitting between the $\lvert FX\rangle$ and $\lvert FY\rangle$ dark exciton states in absence of an external magnetic field
$\hbar\Omega_Z$ -	Zeeman splitting of the bright exciton in the Faraday magnetic field
$\hbar\Omega_{FZ}$ -	Zeeman splitting of the dark exciton in the Faraday magnetic field
$\mathbf{\Omega}_A$ -	direction of the effective magnetic field, $\mathbf{\Omega}_A = (\Omega_X, 0, \Omega_Z)$, for the bright exciton
Ω_A -	Larmor frequency corresponding to the effective magnetic field, $\Omega_A = \sqrt{\Omega_X^2 + \Omega_Z^2}$
$\mathbf{\Omega}_F$ -	direction of the effective magnetic field, $\mathbf{\Omega}_F = (\Omega_{FX}, 0, \Omega_{FZ})$, for the dark exciton
Ω_F -	Larmor frequency corresponding to the effective magnetic field, $\Omega_F = \sqrt{\Omega_{FX}^2 + \Omega_{FZ}^2}$
α -	angle between the individual NPL axis X and laboratory axis x
τ_{sA} -	bright exciton spin relaxation time
τ_{sF} -	dark exciton spin relaxation time
T_A -	bright exciton pseudospin lifetime, $T_A^{-1} = \tau_A^{-1} + \tau_{sA}^{-1}$
T_F -	dark exciton pseudospin lifetime, $T_F^{-1} = \tau_F^{-1} + \tau_{sF}^{-1}$
